# Sho1 and Msb2 Play Complementary but Distinct Roles in Stress Responses, Sexual Differentiation, and Pathogenicity of *Cryptococcus neoformans*

**DOI:** 10.3389/fmicb.2018.02958

**Published:** 2018-12-04

**Authors:** Yee-Seul So, Juyeong Jang, Goun Park, Jintao Xu, Michal A. Olszewski, Yong-Sun Bahn

**Affiliations:** ^1^Department of Biotechnology, College of Life Science and Biotechnology, Yonsei University, Seoul, South Korea; ^2^Division of Pulmonary and Critical Care Medicine, Department of Internal Medicine, University of Michigan Medical School, Ann Arbor, MI, United States; ^3^VA Medical Center Ann Arbor Research Service, Ann Arbor, MI, United States

**Keywords:** HOG, mucin, *C. neoformans*, mating, osmotic stress

## Abstract

The high-osmolarity glycerol response (HOG) pathway is pivotal in environmental stress response, differentiation, and virulence of *Cryptococcus neoformans*, which causes fatal meningoencephalitis. A putative membrane sensor protein, Sho1, has been postulated to regulate HOG pathway, but its regulatory mechanism remains elusive. In this study, we characterized the function of Sho1 with relation to the HOG pathway in *C. neoformans*. Sho1 played minor roles in osmoresistance, thermotolerance, and maintenance of membrane integrity mainly in a HOG-independent manner. However, it was dispensable for cryostress resistance, primarily mediated through the HOG pathway. A mucinlike transmembrane (TM) protein, Msb2, which interacts with Sho1 in *Saccharomyces cerevisiae*, was identified in *C. neoformans*, but found not to interact with Sho1. *MSB2* codeletion with *SHO1* further decreased osmoresistance and membrane integrity, but not thermotolerance, of *sho1*Δ mutant, indicating that both factors play to some level redundant but also discrete roles in *C. neoformans*. Sho1 and Msb2 played redundant roles in promoting the filamentous growth in sexual differentiation in a Cpk1-independent manner, in contrast to the inhibitory effect of the HOG pathway in the process. Both factors also played redundant roles in maintaining cell wall integrity in the absence of Mpk1. Finally, Sho1 and Msb2 play distinct but complementary roles in the pulmonary virulence of *C. neoformans*. Overall, Sho1 and Msb2 play complementary but distinct roles in stress response, differentiation, and pathogenicity of *C. neoformans*.

## Introduction

The high-osmolarity glycerol response (HOG) pathway is a multifunctional signal transduction pathway in pathogenic yeast, *Cryptococcus neoformans*, involved in sensing, responding, and adapting to a plethora of environmental cues, production of virulence factors (e.g., capsule and melanin), and ergosterol biosynthesis (Bahn et al., 2005; Bahn, 2008; Jung and Bahn, 2009; Ko et al., 2009; Bahn and Jung, 2013). The central components of the HOG pathway include the mitogen-activated protein kinase (MAPK) Hog1 (Bahn et al., 2005) and its two upstream kinases, the MAPK kinase (MAPKK) Pbs2 and the MAPKK kinase (MAPKKK) Ssk2 (Bahn et al., 2005, 2007). Hog1 transcriptionally activates various stress-defense genes through multiple transcription factors, while in turn, Hog1 is tightly regulated to prevent its detrimental overactivation (Kruppa and Calderone, 2006; Hohmann et al., 2007; Bahn, 2008; Bahn and Jung, 2013).

The Ssk2/Pbs2/Hog1 MAPK module is primarily activated by the two-component-like phosphorelay system, which comprises two response regulators (Ssk1 and Skn7), a single phosphotransfer protein (Ypd1), and seven putative hybrid histidine kinases (Tco1–7) in *C. neoformans* (Bahn et al., 2006; Lee et al., 2011). However, evidence suggests that the phosphorelay system might not be the only upstream regulator of the HOG pathway in *C. neoformans*. First, the *ssk1*Δ mutant is phenotypically similar, but not equivalent, to the *hog1*Δ mutant, whereas the *pbs2*Δ and *ssk2*Δ mutants are almost phenotypically identical to the *hog1*Δ mutant (Bahn et al., 2005, 2006, 2007). Second, *SSK1* deletion abolishes basal Hog1 phosphorylation levels but does not prevent Hog1 phosphorylation in response to salt shock (Bahn et al., 2006), indicating that other previously unidentified upstream regulator might exist and phosphorylate Hog1 for its activation.

**FIGURE 1:**
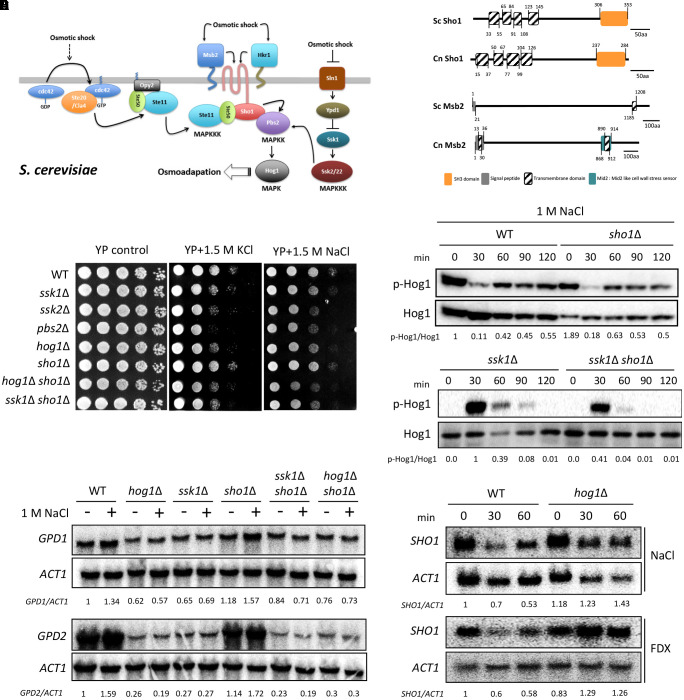
Sho1 regulates the osmotic stress response in Hog1-independent manner. **(A)** Sho1 and Sln1 branches of the HOG pathway induced by the hyperosmolarity in *Saccharomyces cerevisiae*. **(B)** The domain structure of Sho1 and Msb2 proteins in *C. neoformans* and *S. cerevisiae*. The protein domains were analyzed using the Pfam protein domain analysis (http://pfam.xfam.org). The SH3 domain, signal peptide, transmembrane domains, and Mid2 domain are marked. **(C)** Wild-type (WT; H99), *ssk1*Δ (YSB261), *ssk2*Δ (YSB264), *pbs2*Δ (YSB123), *hog1*Δ (YSB64), *sho1*Δ (YSB1719), *hog1*Δ *sho1*Δ (YSB2268), and *ssk1*Δ *sho1*Δ (YSB2253) strains were grown overnight at 30°C in the liquid yeast extract–peptone–dextrose (YPD) medium. The strains were 10-fold serially diluted (1–10^4^ dilutions) and spotted on the YP medium containing the 1.5-M concentration of NaCl or KCl. This spot assay was repeated more than three times and one representative image was shown here. **(D)** Strains were grown to the mid-logarithmic phase and exposed to 1-M NaCl for the indicated times. Total protein extracts were prepared for the western blot analysis. Hog1 phosphorylation levels were monitored using anti-P-p38 antibody. The blot was stripped and used for detection of Hog1 with a polyclonal anti-Hog1 antibody as a loading control. These western blot analyses were repeated twice and one representative result was shown here. **(E,F)** Each strain grown to the mid-logarithmic phase was further incubated in YPD medium containing 1-M NaCl for 30 min **(E)** or YPD medium containing 1-M NaCl or 2-μg/mL fludioxonil (FDX) for the indicated time **(F)**. Total RNAs were prepared for the northern blot analysis. Each membrane was hybridized with the gene-specific probe. The relative expression levels of *GPD1*, *GPD2*, and *SHO1* were quantitatively measured using a PhosphorImager after normalization with *ACT1* expression levels. These northern blot analyses were repeated twice and one representative result was shown here.

One upstream signaling branch potentially feeding into the HOG pathway is a Sho1-dependent pathway (Figure 1A). In *Saccharomyces cerevisiae*, Sho1 is a membrane protein, which contains four transmembrane (TM) domains at the N-terminus and an SH3 domain at the C-terminus (Maeda et al., 1995; Figure 1B). Sho1 primarily localizes to the cytoplasmic membrane at the area of polarized growth, such as the bud neck and emerging bud (Raitt et al., 2000; Reiser et al., 2000). Sho1 plays dual roles in yeast osmosensing. First, Sho1 relays osmosensing signals from two mucin-like TM proteins, Msb2 and Hkr1 (O’Rourke and Herskowitz, 2002; Tatebayashi et al., 2007). Both of these upstream osmosensors physically interact with Sho1 through their TM domains (Figure 1A) to generate intracellular signaling through the cytoplasmic domain of Sho1 (Tatebayashi et al., 2007). Second, Sho1 has an adaptor function by recruiting Pbs2 and the Ste11/Ste50 complex through the SH3 domain (Maeda et al., 1995; Zarrinpar et al., 2004; Tatebayashi et al., 2006). Besides Sho1, a type 1 TM protein, Opy2, plays a role in recruiting the Ste50 adaptor to the plasma membrane (Wu et al., 2006; Tatebayashi et al., 2007; Yamamoto et al., 2010). The MAPKKK Ste11 is phosphorylated by two functionally redundant PAK-like kinases, Ste20 and Cla4, which are recruited by the GTP-bound active form of a small GTPase, Cdc42. The activated Ste11/Ste50 subsequently phosphorylates Pbs2 (Raitt et al., 2000; Tatebayashi et al., 2007). Alternatively, Msb2 itself was proposed to respond to hyperosmotic shock independently of Sho1 and Hkr1 (Tatebayashi et al., 2007). Apart from its role in osmosensing in *S. cerevisiae*, the Sho1 branch is involved in heat-stress response through Hog1 (Winkler et al., 2002). Besides Sho1 itself, Ste20, Ste50, Ste11, and Pbs2 are involved in the heat stress response (Winkler et al., 2002), although the involvement of Msb2 and Hkr2 osmosensors in thermotolerance remains unknown.

The presented map of Sho1-signaling branch in *S. cerevisiae* cannot be directly applied for mapping the HOG pathway in *C. neoformans*. First, Hkr1-like mucin and Opy2-like type 1 TM proteins seem to be missing in the *Cryptococcus* genome. Second, Ste11 and Ste50 are dispensable for most of the Hog1-related phenotypes (Bahn et al., 2007; Jung et al., 2011). Furthermore, deletion of yeast Sho1 ortholog (CNAG_05435) in *C. neoformans*, only weakly decreases thermotolerance but increases *C. neoformans* capsule production (Kim et al., 2015), which could imply that some or all of these effects might be HOG-independent. Finally, while the *sho1*Δ mutant is as virulent as the wild-type (WT) strain in a murine model of systemic cryptococcosis (Kim et al., 2015), Sho1 contributes to the fungal virulence by promoting non-protective Th2 immune response to organism inhaled into the lungs (Malachowski et al., 2016), suggesting a complex relationship between these pathways and virulence. Thus, it remains elusive whether the Sho1-like signaling branch regulates the HOG pathway or else, plays a distinct role in *C. neoformans* and how these signaling circuits (and their crosstalk) affect cryptococcal fitness and virulence.

This study aimed to further examine if the regulatory crosstalk exists between Sho1 and the HOG-signaling pathways and how they functionally relate to the newly identified Msb2-like mucin-TM protein (CNAG_01421) in *C. neoformans.*

## Materials and Methods

### Strain and Media

Supplementary Tables S1, S2 list the strains and primers used in this study. We cultured *C. neoformans* strains in the yeast extract–peptone–dextrose (YPD) medium. Agar-based Dulbecco modified Eagle medium was prepared for the capsule production by combining filter-sterilized 2× DME liquid medium (pH 7.2; Invitrogen Corp.) with autoclaved 2% agar solution. In addition, the melanin production was assessed on Niger seed medium containing a different concentration of glucose.

### Disruption of the *SHO1* and *MSB2* Genes

The *SHO1* and *MSB2* genes were deleted in *C. neoformans* serotype A strain H99 (*MAT*α) as follows. The disruption cassettes were generated by first- and second-round PCR with the primers listed in Supplementary Table S2 using a split marker/double-joint PCR strategy that has been reported previously (Kim et al., 2009). PCR amplifications were performed using the Ex-Taq polymerase (TAKARA). Each disruption construct was purified using the Gel SV kit (Geneall), coated on to gold microcarrier beads [0.6-μm (Bio-Rad)] and introduced into the strain H99 by biolistic transformation. Transformants were selected on YPD-containing nourseothricin, G418, or hygromycin B. The *sho1*Δ and *msb2*Δ mutant strains were confirmed by diagnostic PCR and Southern blot analysis (Supplementary Figure S1).

### Northern Blot Analysis

Each strain was grown in 50-mL YPD medium at 30°C for 16 h. Then, the overnight culture was inoculated into fresh YPD medium and, then, incubated for about 4 h at 30°C to the optical density at 600 nm (OD600) of 0.6. A sample of the liquid culture (50 mL) was taken at each stress time point, frozen in liquid nitrogen, and lyophilized. The total RNAs were isolated with the Ribo-Ex (Geneall). Furthermore, northern blotting was performed on 10 μg of RNA.

### Western Blot Analysis for the Hog1 and Cpk1 Phosphorylation

Each strain was grown in 50-mL YPD medium at 30°C for 16 h. Then, the overnight culture was inoculated into fresh YPD medium and, then, incubated for about 4 h at 30°C to the OD600 of 0.6. A 50 mL of the liquid culture was used at each stress time point. At various time points after the stress, 50 mL of cell suspension was mixed with equal volume of ice-cold stop solution (0.9% NaCl, 1 mM NaN_3_, 10 mM EDTA, and 50 mM NaF). The cells were harvested at 3000 rpm at 4°C for 5 min and, then, washed once in ice-cold stop solution. The cell pellet was resuspended in the lysis buffer (50-mM Tris–HCl pH 7.5, 1% sodium deoxycholate, 5-mM sodium pyrophosphate, 10-nM sodium orthovanadate, 50-mM NaF, 0.1% SDS, and 1% Triton X-100) containing protease inhibitor cocktail (Calbiochem) and disrupted with 0.5-mm zirconia/silica beads (BioSpec Products, Inc.). After collecting the cell lysates, protein concentrations were determined using the Pierce BCA Protein Assay Kit (Thermo Scientific), and an equal amount of protein was loaded into a 10% SDS-PAGE gel and transferred to Immunoblot PVDF membrane (Bio-Rad). For detecting the phosphorylated forms of Hog1, we used phospho-p38 MAPK antibody (Cell Signaling Technology). In addition, anti-Hog1 antibody (Santa Cruz Biotechnology, SC-2004) was used as a loading control. Secondary antibody used was goat anti-rabbit immunoglobulin G peroxidase-conjugated (Santa Cruz Biotechnology, SC-2004) and the blot was developed using the ECL solution.

### The Assay of Sensitivity to Various Stresses

Cells were incubated in 2-mL YPD medium overnight at 30°C, serially diluted (1–10^4^ dilutions) in distilled water and spotted (3 μL) onto a solid YPD medium containing various concentrations of stress reagents. Each plate was incubated for 2–5 days and photographed during the incubation period. Then, cells were spotted on YPD medium containing an indicated concentration of sodium dodecyl sulfate (SDS), Congo red (CR), and calcofluor white (CFW) to test the membrane and cell-wall integrity. Next, cells were spotted on YPD medium containing the indicated concentration of diamide, menadione, *tert*-butyl hydroperoxide, and hydrogen peroxide to assess oxidative stress. Furthermore, cells were spotted on YPD medium containing the indicated concentration of polyene (amphotericin B), azole (fluconazole, ketoconazole), flucytosine, and fludioxonil to test the antifungal drug sensitivity. To test the temperature sensitivity, plates were incubated at 30, 37, and 40°C.

### The Freeze–Thaw Assay

Each strain was grown in 50 mL YPD medium at 30°C for 16 h. Next, the overnight culture was inoculated into fresh YPD medium and, then, incubated for about 4 h at 30°C to the OD600 of 0.6. The cells were frozen in liquid nitrogen for 1 min and, then, melted in a 30°C water bath for 15 min; this process was repeated. These cells were serially diluted (1–10^4^ dilutions) and spotted on solid YPD medium. Each plate was incubated for 2–4 days and photographed.

### Sho1 and Msb2 Localization Study

The *sho1*Δ*::SHO1-GFP* complemented strain was constructed as follows (Supplementary Figure S1). The *SHO1* 5′-untranslated region (UTR) and open reading frame (ORF) was amplified using PCR and cloned into pTOP vector (Enzynomics) and sequenced. The *SHO1* gene insert was subcloned into the pJAF12, which contains neomycin/G418-resistant marker, generating the plasmid pJAF12-SHO1. The *GFP* and *SHO1* 3′*-*UTR regions were also amplified and fused by PCR with the primers listed in Supplementary Table S2. The *GFP*-*SHO1* 3′-UTR fusion PCR product was cloned into the pTOP vector and sequenced, generating the plasmid pTOP_GFP-SHO1 3′UTR. Then the *GFP*-*SHO1* 3′-UTR insert was subcloned into the pJAF12_SHO1 to generate pJAF12_SHO1-GFP. The pJAF12_SHO1-GFP was linearized by HindIII and biolistically introduced into the *sho1*Δ mutant strain (YSB1719). Furthermore, diagnostic PCR and phenotypic analyses were performed to confirm the targeted or ectopic reintegration of the *SHO1* gene. To construct the *MSB2-mCherry* and *sho1*Δ*::SHO1-GFP* *MSB2*-*mCherry* strains, the *MSB2-mCherry* cassette for chromosomal Msb2 C-terminal tagging was generated using the primers listed in Supplementary Table S2 by a split marker/double-joint PCR strategy that has been reported previously (Kim et al., 2009). The *MSB2-mCherry* cassettes were delivered into the H99S and *sho1*Δ*::SHO1-GFP* strains (YSB2753) by biolistic transformation. The tagged strains were confirmed by Southern blot and phenotypic analysis (Supplementary Figures S1, S2). The *sho1*Δ*::SHO1-GFP*, *MSB2*-*mCherry*, and *sho1*Δ*::SHO1-GFP* *MSB2*-*mCherry* strains were incubated overnight at 30°C in YPD medium to observe the Sho1 and Msb2 protein localization. Furthermore, the cells were fixed and visualized by a Nikon Eclipse Ti microscope.

### Coimmunoprecipitation and Immunoblotting

The *MSB2-4*×*FLAG* and *SHO1-6*×*HA* cassettes for chromosomal Msb2 and Sho1 C-terminal tagging, respectively, were generated using primers listed in Supplementary Table S2 by a split marker/double-joint PCR strategy (Supplementary Figure S1). The *MSB2-4*×*FLAG* tagging cassettes were delivered into the H99S and *SHO1-6×HA* (YSB3593) by biolistic transformation. Proper construction of each tagged strain was confirmed by Southern blot and phenotypic analysis (Supplementary Figures S1, S2). The *MSB2-4*×*FLAG*, *SHO1-6*×*HA*, and *MSB2-4*×*FLAG* *SHO1-6*×*HA* strains were incubated in YPD liquid medium overnight at 30°C. The overnight culture was inoculated into 100 mL of fresh YPD liquid medium and, then, incubated at 30°C until the OD600 reached approximately 0.8. In addition, whole cell lysates of strains were prepared according to the method described above. After adding an anti-HA antibody (Sigma-Aldrich), the whole-cell lysates were rotated overnight at 4°C. Next, sepharose protein G beads (GE Healthcare Life Sciences) were added to the whole-cell lysates and rotated for 6 h at 4°C. To remove the unbound proteins, the mixture was centrifuged, and the pellet was washed six times with lysis buffer. The proteins bound to the beads were eluted with the SDS sample buffer (50-mM Tris–HCl, 2% SDS, 10% glycerol, and 0.01% mercaptoethanol) and detected by immunoblotting with anti-FLAG (Santa Cruz Biotechnology) and anti-HA (Roche) antibodies.

### The Assay for Capsule Production

Each strain was incubated overnight in YPD medium at 30°C. The cells were spotted onto a solid DME medium, and further incubated for 2 days at 37°C. After incubation, the capsule was visualized with India Ink (Remel) staining and observed with an Olympus BX51 microscope equipped with SPOT Insight digital camera (Diagnostic Instrument Inc.). Furthermore, diameters of the capsule and cell body were measured for the quantitative analysis of the capsule production.

### Mating, Cell Fusion, and Pheromone Gene Expression Assay

For analyzing mating phenotypes opposite mating type (*MATα* and *MAT**a***) cells were cultured in YPD medium at 30°C for 16 h and equal concentration of cells (10^7^ cells/mL) were mixed, spotted onto V8 mating media (pH 5), and incubated in the dark at room temperature for 1–2 weeks. The filamentous growth was monitored and photographed using an Olympus BX51 microscope equipped with a SPOT Insight digital camera. For the cell fusion assay, the concentration of cells was adjusted to 10^7^ cells/mL with phosphate-buffered saline. Each *MAT*α and *MAT**a*** strain was mixed in an equal volume, spotted onto a V8 medium, and incubated in the dark at room temperature for 24 h. Then, the cells were scraped, resuspended in 1-mL distilled water, and spread onto YPD medium containing both nourseothricin (100 μg/mL) and G418 (50 μg/mL). The plates were further incubated at 30°C, and the number of colonies was counted. For monitoring the pheromone gene expression, the *MAT*α and KN99**a** strains were mixed with an equal concentration of cells (10^8^ cells/mL), spread onto the V8 medium, and incubated in the dark at room temperature for 18 or 24 h. Then, cells were scraped, pelleted, frozen in liquid nitrogen, and lyophilized overnight for the total RNA isolation, followed by the northern blot analysis with the specific mating pheromone gene (*MFα1*)-specific probe.

### Mice

In total, 80 mice have been used for this study. BALB/c mice (8 weeks old of both sexes) were obtained from The Jackson Laboratory (Bar Harbor, ME, United States) and housed under specific pathogen-free conditions in the Animal Care Facility at the Veterans Affairs Ann Arbor Healthcare System, with food and water provided *ad* *libitum* and with daily veterinary oversite. All experiments were approved by the Veterans Administration Healthcare System’s Institutional Animal Care and Use Committee. Mice were under careful post-procedural monitoring for any potential side effects, according to the US Federal and the institutional animal care guidelines and SOPs.

### Intratracheal Inoculation of *C. neoformans*

For infection with *C. neoformans*, cryptococcal strains were grown at 37°C in Sabouraud dextrose broth (Difco, Detroit, MI, United States), washed with PBS, enumerated under the microscope and diluted with PBS to the final concertation 5 × 10^5^ cells/mL. Mice were anesthetized via intraperitoneal injection of ketamine (100 mg/kg body weight) with xylazine (6.8 mg/kg). A small incision on the skin of mouse neck was made to expose the trachea. Thirty microliters (10^4^ CFU) of *C. neoformans* were injected into the lungs intratracheally using a 30-gauge needle attached to a 1-mL tuberculin syringe. After inoculation, the skin was closed with cyanoacrylate adhesive and the mice were monitored during recovery from the anesthesia and daily for potential development of any adverse post-procedural effects and pre-established endpoint criteria. All animals fully recovered from the procedure and showed no apparent symptoms within the studied time course of infection.

### Lung Fungal Burden Assay

For determination of fungal burden, dissected lungs were homogenized in 2 mL of sterile water. Small aliquots of digested lungs and series of 10-fold dilutions were plated on Sabouraud dextrose agar plates in duplicate 10-μL aliquots. *C. neoformans* colonies were counted 48 h later and the number of CFU was calculated on a per-organ basis.

## Results

### Sho1 Plays a Ssk1/Hog1-Independent Role in Osmosensing and Response in *C. neoformans*

In *S. cerevisiae*, the well-established function of Sho1 is to mediate osmosensing signals generated by two mucin-like TM proteins, Msb2 and Hkr1 (Tatebayashi et al., 2007; Figures 1A,B). Hence, we first addressed whether *C. neoformans* Sho1 plays any role in sensing or mediating osmotic shock signals. We used the *sho1*Δ mutant, which we constructed previously (Kim et al., 2015), and here additionally constructed the *sho1*Δ mutants in *hog1*Δ and *ssk1*Δ strain backgrounds to investigate an epistatic correlation between Sho1 and Hog1 or Ssk1.

First, we determined the osmosensitivity of the *sho1*Δ mutant compared with *ssk1*Δ, *ssk2*Δ, *pbs2*Δ, and *hog1*Δ mutants. The *sho1*Δ mutant was as resistant to 1.5-M NaCl or KCl as the WT strain, whereas *ssk2*Δ, *pbs2*Δ, and *hog1*Δ mutants were hypersensitive to the osmotic stresses (Figure 1C). As reported previously (Bahn et al., 2006), the *ssk1*Δ mutant also showed an increased sensitivity to the osmotic stresses, albeit to a lesser extent than the *hog1*Δ mutant (Figure 1C). In *C. neoformans*, Hog1 is markedly phosphorylated under the unstressed condition and starts to be dephosphorylated in response to osmotic shock (Bahn et al., 2005; Figure 1D). Hog1 dephosphorylation patterns in the *sho1*Δ mutant were almost identical to those of the WT strain in response to osmotic shock (1 M NaCl). In the *ssk1*Δ mutant, Hog1 was unphosphorylated but started to be phosphorylated in response to osmotic shock, which was consistent with our previous finding (Bahn et al., 2006), and these Hog1 phosphorylation patterns were identical in the *sho1*Δ *ssk1*Δ mutant (Figure 1D). These data strongly supported that Sho1 is not the unknown signaling component that we had expected to trigger the Hog1 phosphorylation in the absence of Ssk1. This was further solidified by the finding that the expressions of two genes, glycerol-3-phosphate dehydrogenase 1 and 2 (*GPD1* and *GPD2*, respectively) (Ko et al., 2009), induced by osmotic stress in a Hog1-dependent manner were also unaltered in the *sho1*Δ mutant (Figure 1E). Furthermore, the basal expression level of *GPD1* and *GPD2* was markedly decreased in the *hog1*Δ and *ssk1*Δ mutants but not in the *sho1*Δ mutant (Figure 1E). Collectively these data provide strong evidence that Sho1 is not required for the activation of Hog1 in response to osmotic shock.

To further examine mutual relationship between Hog1, Ssk1, and Sho1 in osmosensing, responses of *sho1*Δ *hog1*Δ and *sho1*Δ *ssk1*Δ double mutants to the osmotic stress have been tested. The *sho1*Δ *hog1*Δ double-mutant showed higher susceptibility to osmotic stress (1.5 M NaCl in particular) than the *hog1*Δ mutant, indicating that *SHO1* deletion further compromised osmotolerance of the *hog1*Δ mutant (Figure 1C). The *sho1*Δ *ssk1*Δ double-mutant was as osmosensitive as the *sho1*Δ *hog1*Δ mutant and more sensitive than each single *ssk1*Δ or *hog1*Δ mutant (Figure 1C), supporting that these molecules, while working independently, were all contributing to the osmotic stress response. The subsequent northern blot analysis revealed that *SHO1* expression levels were decreased by osmotic shock or another Hog1-signaling activator, fludioxonil, in the WT *C. neoformans* (Figure 1F). However, such osmotic-shock- or fludioxonil-dependent *SHO1* reduction was not observed in the *hog1*Δ mutant (Figure 1F), indicating that the *SHO1* expression might be maintained in the absence of Hog1 in response to osmotic shock or fludioxonil treatment, perhaps because of a compensatory effect or that Hog1 is somehow involved in *SHO1* suppression. Overall, Sho1 plays a role in osmosensing in parallel with the Ssk1-dependent and Hog1-dependent signaling branches.

### Sho1 Controls Thermotolerance in a Hog1-Independent Manner

**FIGURE 2:**
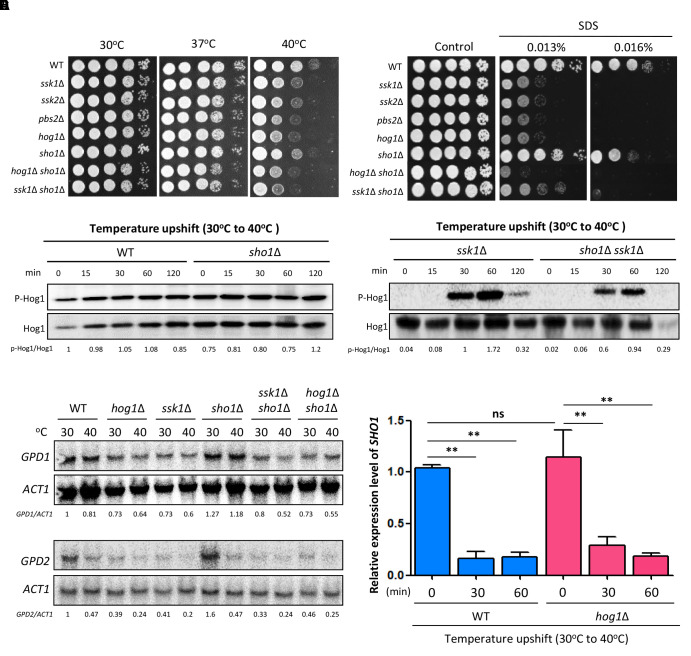
Sho1 controls thermotolerance of *C. neoformans* in the Hog1-independent manner. **(A)** Wild-type (WT; H99), *ssk1*Δ (YSB261), *ssk2*Δ (YSB264), *pbs2*Δ (YSB123), *hog1*Δ (YSB64), *sho1*Δ (YSB1719), *hog1*Δ *sho1*Δ (YSB2268), and *ssk1*Δ *sho1*Δ (YSB2253) strains were grown overnight at 30°C in liquid YPD medium. To test the thermosensitivity, cells were 10-fold serially diluted (1**–**10^4^ dilutions), spotted on solid YPD medium, and further incubated at 37 or 40°C. The spot assay was repeated more than three times and the one representative image was shown here. **(B)** To test the sodium dodecyl sulfate (SDS) sensitivity, cells were 10-fold serially diluted (1**–**10^4^ dilutions) and spotted on YPD medium containing the indicated concentration of SDS. The spot assay was repeated more than three times and one representative image was shown here. **(C)** Strains were grown to the mid-logarithmic phase and further incubated at 40°C for the indicated time. Total protein extracts were prepared for the western blot analysis. Hog1 phosphorylation were monitored using anti-P-p38 antibody. The blot was stripped and used for detection of Hog1 with a polyclonal anti-Hog1 antibody as a loading control. These western blot analyses were repeated twice and one representative result was shown here. **(D)** Each strain was incubated at 30 or 40°C for 30 min. The northern blot analysis was performed with total RNAs isolated from each strain. Each membrane was hybridized with the gene-specific probe. The relative expression levels of *GPD1* and *GPD2* were quantitatively measured using a PhosphorImager after normalization with *ACT1* expression levels (*GPD1/ACT1* and *GPD2/ACT1*). These northern blot analyses were repeated twice and one representative result was shown here. **(E)** The expression levels of *SHO1* was verified by qRT-PCR analysis using cDNA synthesized from the total RNA isolated from WT (H99) and *hog1*Δ (YSB64) upon the temperature upshift from 30 to 40°C. Three independent biological experiments with triplicate technical replicates were performed. Error bars, SEM. Statistical significance of difference was determined by the one-way analysis of variance with the Bonferroni’s multiple-comparison test (^∗∗^*P* < 0.01; ns, not-significant).

Sho1 in *S. cerevisiae* is reported to sense and respond to a temperature upshift, making it likely to be involved in thermotolerance in *C. neoformans*. Consistently, the *sho1*Δ mutant showed a weak growth defect upon temperature upshift (30–40°C) compared with the WT strain, albeit to a lesser extent than the *hog1*Δ mutant (Figure 2A). Complementation of the *sho1*Δ mutant with the WT *SHO1* gene tagged with an HA epitope or a *GFP* gene restored the WT level of thermotolerance (Supplementary Figure S2), corroborating the role of Sho1 in thermotolerance of *C. neoformans.* *SHO1* deletion marginally increased the thermosensitivity of the *hog1*Δ or *ssk1*Δ mutant (Figure 2A), suggesting that Sho1 might work in parallel with Ssk1 and Hog1 helping to overcome the effects of thermal stress. We next assessed whether the decreased thermotolerance observed in the *sho1*Δ mutant is associated with the cell membrane stability. The *sho1*Δ mutant showed increased sensitivity to SDS (cell membrane destabilizer), albeit to a lesser extent that the *hog1*Δ and *ssk1*Δ mutants (Figure 2B), providing a clue that Sho1 modestly contributes to the cell membrane stability, but again these effects do not resemble the strong effects of Hog1 and Ssk1 in this area.

To further assess whether Sho1 contributes to thermotolerance through Hog1, we monitored Hog1 phosphorylation patterns in the WT strain and the *sho1*Δ mutants during the temperature upshift. Upon the temperature upshift, Hog1 phosphorylation levels did not change in the WT strain (Figure 2C). Similar to the case under osmotic shock, Hog1 in the *ssk1*Δ mutant became highly phosphorylated by the temperature upshift (Figure 2C), indicating that the Hog1 phosphorylation is affected by the temperature upshift in the absence of Ssk1. *SHO1* deletion did not markedly change Hog1 phosphorylation patterns in the WT and *ssk1*Δ mutant, although Hog1 phosphorylation induction in the *ssk1*Δ mutant was weakly reduced by *SHO1* deletion (Figure 2C). These results suggest that Sho1 may play a very minor role, if any, in Hog1 regulation and another Sho1-independent signaling branch might exist to activate the Hog1 phosphorylation during the temperature upshift.

The finding that Hog1 underwent similar phosphorylation patterns in the *ssk1*Δ mutant during the temperature upshift and the osmotic shock, encouraged us to address whether these two stressors induced similar cellular responses. We monitored *GPD1* and *GPD2* expression during the temperature upshift. In contrast to the osmotic shock conditions, neither *GPD1* nor *GDP2* has been upregulated while the *GPD2* expression was substantially diminished upon the temperature upshift in both WT and *sho1*Δ mutants (Figure 2D). These outcomes imply that the two stresses trigger distinct cellular responses and that Sho1 is not involved in suppressing *GPD2* during heat shock response. In addition, we monitored *SHO1* expression patterns during the temperature upshift. Similar to the case of the osmotic stress response, the *SHO1* expression was markedly decreased upon the temperature upshift despite its beneficial role in thermotolerance (Figure 2E). Such *SHO1* reduction was equally observed in the *hog1*Δ mutant, suggesting that Sho1 is downregulated during thermal stress and this Sho1 regulation is Hog1 independent. Overall, our data demonstrate that Sho1 contributes to *C. neoformans* thermotolerance mainly in both Hog1- and Ssk1-independent manner.

### Hog1 Governs the Cryostress Response in a Ssk1-Dependent, but Sho1-Independent Manner

**FIGURE 3:**
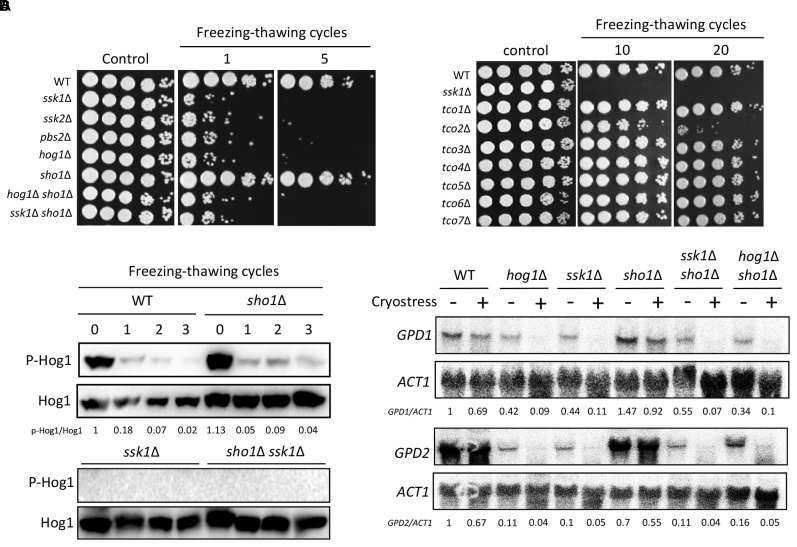
Hog1 governs the cryostress response in a Ssk1-dependent, but Sho1-independent, the manner in *C. neoformans*. **(A,B)** Wild-type (WT; H99), *ssk1*Δ (YSB261), *ssk2*Δ (YSB264), *pbs2*Δ (YSB123), *hog1*Δ (YSB64), *sho1*Δ (YSB1719), *hog1*Δ *sho1*Δ (YSB2268), *ssk1*Δ *sho1*Δ (YSB2253), *tco1*Δ (YSB278), *tco2*Δ (YSB281), *tco3*Δ (YSB284), *tco4*Δ (YSB417), *tco5*Δ (YSB286), *tco6*Δ (YSB2469), and *tco7*Δ (YSB348) strains were grown overnight at 30°C in liquid YPD medium. The cells were frozen in liquid nitrogen for 1 min and then melted in a 30°C water bath for 15 min; this process was repeated as the indicated number of the cycle in the figure. After that, cells were 10-fold serially diluted (1–10^4^ dilutions) and spotted 3 μL on YPD medium. These experiments were repeated more than three times and the one representative image was shown here. **(C)** WT (H99) and *sho1*Δ (YSB1719) strains were grown to the mid-logarithmic phase and, then, repeated the freezing and thawing process by cycles indicated and total protein extracts were prepared for the western blot analysis. These western blot analyses were repeated twice and one representative result was shown here. **(D)** The northern blot analysis was performed with the total RNAs isolated from each strain by repeating two freezing and thawing cycles. Each membrane was hybridized with the gene-specific probe. The relative expression levels of *GPD1* and *GPD2* were quantitatively measured using a PhosphorImager after normalization with *ACT1* expression levels (*GPD1/ACT1* and *GPD2/ACT1*). These northern blot analyses were repeated twice and one representative result was shown here.

Given the role of the Sho1-signaling and Ssk1/Hog1-signaling pathways in the thermotolerance of *C. neoformans*, we investigated whether the two pathways were also involved in the cellular adjustment to cold or freezing temperature. Thus, we assessed the cell viability of each mutant after repeated freezing and thawing process (cryostress). Interestingly, the *ssk1*Δ, *ssk2*Δ, *pbs2*Δ, and *hog1*Δ mutants showed extreme sensitivity to cryostress (Figure 3A), strongly suggesting that the Ssk2/Pbs2/Hog1-signaling module is essential for the cryostress response and adaptation. Upstream of the MAPK module, the *ssk1*Δ mutant showed the similar level of the cryostress sensitivity (Figure 3A). In addition, upstream of the Ssk1 response regulator, seven hybrid histidine kinases (Tco1–7) have been reported, among which Tco1 and Toc2 play redundant and distinct roles in regulating Ssk1 (Bahn et al., 2006). For the cryostress response, only the *tco2*Δ mutants showed the increased sensitivity, albeit to a much lesser extent than the *hog1*Δ mutant (Figure 3B). These findings suggest that Tco2 might play positive roles in regulating Ssk1 during the cryostress response in *C. neoformans*.

By contrast, the *sho1*Δ mutant was as resistant to cryostress as the WT strain (Figure 3A), indicating that Sho1 is dispensable for the cryostress resistance. Supporting this finding, Hog1 was rapidly dephosphorylated after single freezing–thawing cycle in both WT and *sho1*Δ strains (Figure 3C). Conversely, Hog1 was not phosphorylated in the *ssk1*Δ or *ssk1*Δ *sho1*Δ mutant, unlike in the case of osmosensing and thermotolerance (Figure 3C), suggesting that the Ssk1 branch is the only upstream signaling pathway for the Hog1 activation during cryostress response and adaptation. A prior study suggested that *S. cerevisiae* responds to and adapts to cryostress by activating Hog1 and inducing the *GPD1* and *GPD2* expression to increase the intracellular glycerol content (Hayashi and Maeda, 2006). However, we observed that cryostress failed to induce the *GPD1* and *GPD2* expression in *C. neoformans* (Figure 3D), indicating that the Hog1-dependent cryostress resistance might not result from the increased *GPD1* and *GPD2* expression. Overall, these findings suggested that the Ssk1/Hog1-signaling pathway, but not a Sho1-signaling pathway, promotes the cryostress resistance in *C. neoformans*, further supporting that Sho1 and Hog1 pathways work independently, supporting different types of cellular responses.

### Identification of a Msb2-Like Mucin TM Protein in *C. neoformans*

Having determined that Sho1 function is not significantly related to Hog1 pathway regulation, we sought to establish if it is linked to other proteins known to interact with Sho1 in yeasts. In *S. cerevisiae*, two mucin-like TM proteins Msb2 and Hkr1 physically interact with Sho1 (Tatebayashi et al., 2007; Figure 1A). To explore other mechanistic links with the Sho1-signaling pathway in *C. neoformans*, we performed searches for Msb2 and Hkr1 orthologs. We found that *C. neoformans* has a single mucin-like TM protein (CNAG_01421), which is more homologous to Msb2 (score: 43.9, e-value: 3.9e-06) than to Hkr1. Like Msb2 from *S. cerevisiae*, CNAG_01421 protein contains a Mid2 domain at the C-terminus with a TM region functioning in yeast as the mechanosensor of cell-wall stress (Figure 1B). Considering these similarities, we designated CNAG_01421 as cryptococcal Msb2.

**FIGURE 4:**
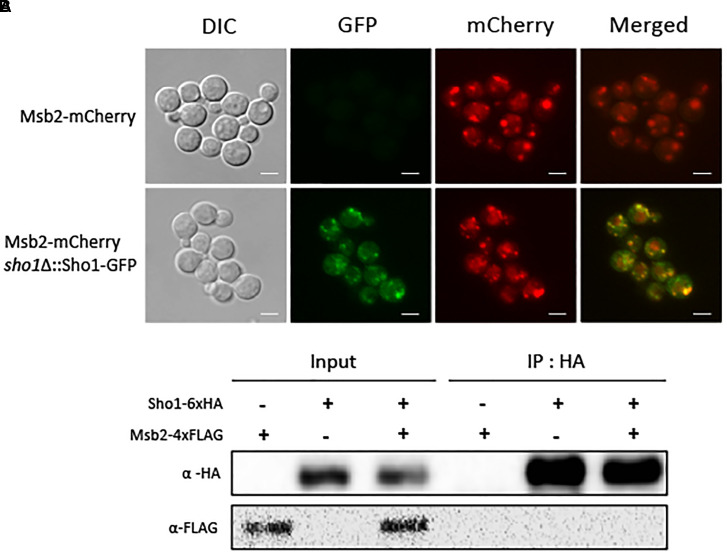
Both Sho1 and Msb2 proteins appeared to be localized to the cell periphery, but Sho1 and Msb2 did not physically interact with each other. **(A)** To determine the localization of Sho1 and Msb2, the *sho1*Δ*::SHO1-GFP* (YSB2753), *MSB2-mCherry* (YSB4128), and *sho1*Δ*::SHO1-GFP* *MSB2-mCherry* (YSB4132) strains were grown overnight at 30°C in liquid YPD medium. The cells were fixed and visualized by fluorescence microscopy. Scale bar, 10 μm. **(B)** The Sho1-6×HA protein was immunoprecipitated with an anti-HA antibody (IP: α-HA), and the Msb2-4×FLAG protein was detected by immunoblotting with an anti-FLAG antibody (IB: α-FLAG). This experiment was repeated twice and one representative result was shown here.

We constructed *C. neoformans* strains expressing *SHO1-GFP*, *MSB2-mCherry*, or both proteins to determine whether *C. neoformans* Sho1 and Msb2 colocalize to the cell membrane. Both Sho1-GFP fusion proteins were confirmed to be functional because complementation of the *SHO1-GFP* allele completely restored WT phenotypes in the *sho1*Δ mutant (Supplementary Figure S2). In addition, the Msb2-mCherry fusion has not resulted in any essential change of function, because the chromosomal tagging of mCherry to the C-terminus of Msb2 in two independently generated constructs have not induce any detectable deviation from the WT phenotypes (Supplementary Figure S2). Both Sho1 and Msb2 proteins appeared to be localized to the cell periphery, although they exist as punctate forms (Figure 4A). Notably, fluorescence signals of Sho1–GFP and Msb2–mCherry overlapped markedly, but not exactly. To address whether Sho1 physically interacts with Msb2, we generated Sho1-6×HA, Msb2-4×FLAG, and Sho1-6×HA Msb2-4×FLAG strains to perform a coimmunoprecipitation experiment. Moreover, the strains were confirmed by genotypic and phenotypic analyses (Supplementary Figures S1, S2). In contrast to Sho1 and Msb2 in *S. cerevisiae*, an interaction between Sho1 and Msb2 could not be supported with this assay, but Sho1 and Msb2 rather colocalized in the *C. neoformans* cell periphery (Figure 4B).

### Msb2 and Sho1 Play Redundant and Distinct Roles in the Stress Response of *C. neoformans*

**FIGURE 5:**
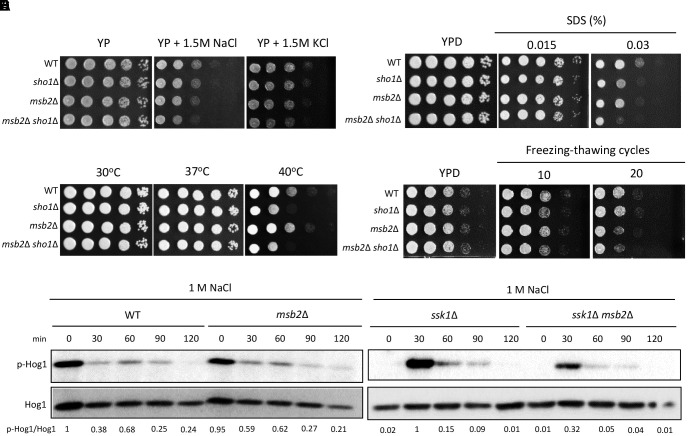
Sho1 and Msb2 are required for the membrane stability. **(A,B)** Wild-type (WT; H99), *sho1*Δ (YSB1719), *msb2*Δ (YSB3191), and *sho1*Δ *msb2*Δ (YSB3605) strains were grown overnight at 30°C in the liquid YPD medium. The strains were 10-fold serially diluted (1–10^4^ dilutions) and spotted 3 μL on YP or YPD medium containing the 1.5-M concentration of NaCl or KCl or SDS. The plates were incubated at 30°C for 2 days and photographed. These spot assays were repeated more than three times and one representative image was shown here. **(C)** Each strain was 10-fold serially diluted (1–10^4^ dilutions), spotted onto solid YPD medium, and further incubated at 30, 37, and 40°C. These spot assays were repeated more than three times and one representative image was shown here. **(D)** Strains were repeatedly frozen and thawed, then 10-fold serially diluted (1–10^4^ dilution), and spotted onto YPD medium. The plates were further incubated for 2–3 days and photographed. This experiment was repeated twice and one representative result was shown here. **(E)** Strains were grown to the mid-logarithmic phase and exposed to 1-M NaCl for the indicated time. The phosphorylation levels of Hog1 were monitored using anti-P-p38 antibody. The blot was stripped and used for detection of Hog1 with polyclonal anti-Hog1 antibody as a loading control. These western blot analyses were repeated twice and one representative result was shown here.

We constructed the *msb2*Δ and *msb2*Δ *sho1*Δ double-mutants in the H99 strain background to establish the function of Msb2 in *C. neoformans.* We first examined the osmosensitivity of the *msb2*Δ mutant to address whether Msb2 acts as an osmosensor. The *msb2*Δ mutant was as resistant to osmotic shock (1.5-M NaCl and KCl) as WT and *sho1*Δ mutant strains (Figure 5A). However, the *msb2*Δ *sho1*Δ mutant showed a higher sensitivity to osmotic shock than WT and each single-mutant strain (Figure 5A), indicating that Msb2 and Sho1 participate in the cryptococcal osmoresistance, but their function is redundant. We further tested the SDS sensitivity of the mutants to establish Sho1- and Msb2-specific roles in maintaining the membrane integrity. The *sho1*Δ and *msb2*Δ mutants showed the increased sensitivity to SDS and the *sho1*Δ *msb2*Δ mutant showed higher sensitivity to SDS than each single mutant (Figure 5B), suggesting that Sho1 and Msb2 play complementary roles in the membrane integrity maintenance.

As both Sho1 and Msb2 are required for the membrane stability, we also assessed the role of both proteins in thermotolerance and cryostress tolerance. Unexpectedly, the *msb2*Δ mutant did not show any thermosensitivity unlike the *sho1*Δ mutant, demonstrating that Msb2 is not required for cryptococcal thermotolerance (Figure 5C). Instead, the *msb2*Δ mutant showed the marginally increased sensitivity to cryostress unlike the *sho1*Δ mutant (Figure 5D). These data collectively demonstrate that cryptococcal Sho1 and Msb2 contribute to distinct mechanism of cell membrane protection, which oppose its damage induced by different factors, e.g., chemical, thermal, and cryostress.

To assess whether Msb2 is involved in Hog1 regulation, we monitored Hog1 phosphorylation patterns in the WT strain and the *msb2*Δ mutants in response to osmotic shock (1 M NaCl). Hog1 undergoes normal dephosphorylation in the *msb2*Δ strain (Figure 5E). In the *ssk1*Δ mutant, *MSB2* deletion did not markedly change Hog1 phosphorylation patterns, but weakly reduced the Hog1 phosphorylation induction level (Figure 2C). All these results suggest that Msb2 does not play a major role, if any, in Hog1 phosphorylation in *C. neoformans*.

### Sho1, but Not Msb2, Represses the Capsule Production

**FIGURE 6:**
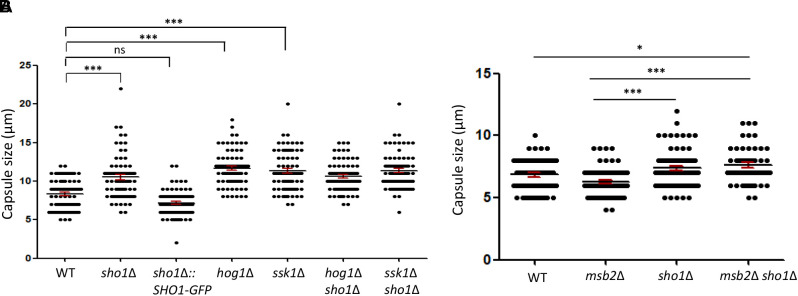
Sho1, but not Msb2, plays a repressive role in the capsule production in *C. neoformans*. **(A,B)** The capsule production was observed microscopically. Wild-type (WT; H99), *sho1*Δ (YSB1719), *sho1*Δ*::SHO1-GFP* (YSB2753), *hog1*Δ (YSB64), *ssk1*Δ (YSB261), *hog1*Δ *sho1*Δ (YSB2268), *ssk1*Δ *sho1*Δ (YSB2253), *msb2*Δ (YSB3191), and *sho1*Δ *msb2*Δ (YSB3605) strains were spotted onto Dulbecco modified Eagle medium, incubated for 2 days at 37°C. Graph of capsule diameter of all the strains. Capsule diameter was determined using the equation [(total diameter) – (cell body diameter)]. The statistical significance is indicated as follows: ns, not-significant; ^∗^*P* < 0.05 and ^∗∗∗^*P* < 0.001.

Previously, we reported that *SHO1* deletion increases the capsule production, but not melanin production, in *C. neoformans* (Kim et al., 2015). Thus, we next addressed whether Msb2 and Sho1 play redundant or distinct roles in the capsule production. The *ssk1*Δ and *hog1*Δ mutants were all enhanced in the capsule production (Figure 6A), as reported previously (Bahn et al., 2005, 2006, 2007). Consistent with previous findings, we observed that the *sho1*Δ mutant showed slightly increased capsule production, albeit to a lesser extent than the *hog1*Δ mutant (Figure 6A). However, the *SHO1* deletion did not further increase the capsule sizes of the *ssk1*Δ and *hog1*Δ mutants (Figure 6A).

In contrast to the suppressive role of Sho1 in the capsule production, Msb2 was dispensable for the capsule production, since *MSB2* deletion did not affect the capsule production (Figure 6B). In addition, the double deletion of *MSB2* did not further increase the enhanced capsule production of the *sho1*Δ mutant (Figure 6B), suggesting that Msb2 does not play a redundant repressive role with Sho1 for the capsule production. Overall, Sho1, but not Msb2, has a repressive role in the capsule production in *C. neoformans*, further supporting that biological functions of Sho1 and Msb2 are distinct.

### Sho1 and Msb2 Play Redundant Roles in the Filamentation Process of *C. neoformans*

**FIGURE 7:**
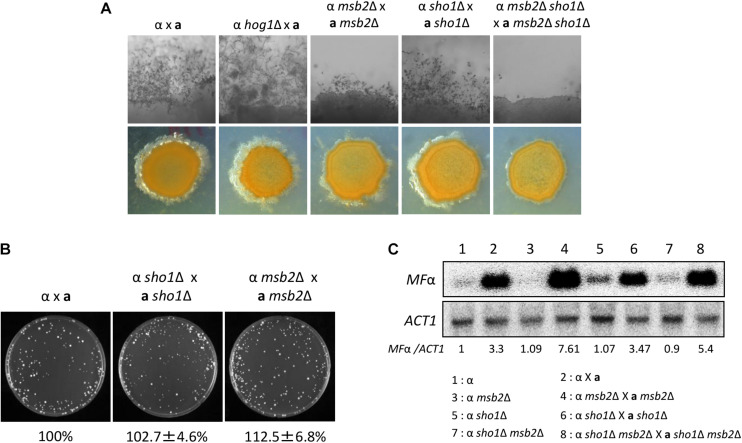
Sho1 and Msb2 play redundant positive roles in the filamentous growth of *C. neoformans*. **(A)** Opposite mating type (*MATα* and *MAT**a***) cells were incubated for 16 h in YPD liquid medium at 30°C. Opposite mating type cells were mixed at equal concentration (10^7^ cells/mL), spotted (5 μL) on V8 medium, and further incubated in the dark at room temperature for 2 weeks. This mating experiment was repeated twice and one representative image was shown here. **(B)** Mixed opposite mating type cells were spotted on V8 medium and incubated for 1 day at room temperature in the dark. After cells were grown on V8 medium, the cells were resuspended in 1-mL dH_2_O and diluted to 1/100. Then, 200 μL of the suspension was spread on YPD medium containing nourseothricin and G418. The plates were further incubated at 30°C and colonies were counted. **(C)** The northern blot analysis was performed with total RNAs from strains grown on V8 medium for 18 h. The northern blot membrane was hybridized with the mating pheromone-gene (*MFα1*)-specific probe. This northern blot analysis was repeated twice and one representative result was shown here.

In *S. cerevisiae*, the Sho1/Msb2-signaling pathway regulates the filamentation process through the Ste11/Ste7/Kss1 MAPK module in response to the partial nutrient deprivation (O’Rourke and Herskowitz, 1998). Sexual differentiation is critical for the generation of infectious spores in *C. neoformans* (Kraus et al., 2003). The HOG pathway is involved in this process by repressing the pheromone production (Bahn et al., 2005). We constructed the *MAT***a** *sho1*Δ, *msb2*Δ, and *msb2*Δ *sho1*Δ mutants in the *MAT***a** KN99**a** strain, which is derived from the *MAT*α H99 strain (Nielsen et al., 2003), to address the role of Sho1 and Msb2 in the mating process. Single deletion of either *SHO1* or *MSB2* did not affect the mating efficiency markedly in both unilateral and bilateral matings (Figure 7A). Moreover, cell fusion normally occurred in the *sho1*Δ and *msb2*Δ single-mutant strains (Figure 7B). Conversely, the double deletion of *SHO1* and *MSB2* severely decreased the filamentous growth (Figure 7A). The decreased filamentous growth was most evident in the bilateral mating set-up (*MAT*α *sho1*Δ *msb2*Δ ×*MAT***a** *sho1*Δ *msb2*Δ), suggesting that Sho1 and Msb2 are required, but play redundant roles in the mating process of *C. neoformans*.

We monitored pheromone expression levels under the unilateral and bilateral mating setup among *sho1*Δ, *msb2*Δ, and *sho1*Δ, *msb2*Δ mutants compared with the WT strain to determine which stage of mating is regulated by Sho1 and Msb2. We observed that the pheromone-gene expression was as markedly induced in the *sho1*Δ, *msb2*Δ, and *sho1*Δ, *msb2*Δ mutants as WT when α cells were cocultured with **a** cells (Figure 7C). These findings suggested that Sho1 and Msb2 play complementary positive roles in the late stage (filamentation), but not the early stage (pheromone expression and cell fusion), of mating in *C. neoformans*.

### The Role of Cpk1, Msb2 and Sho1 in the Cell-Wall Integrity of *C. neoformans*

**FIGURE 8:**
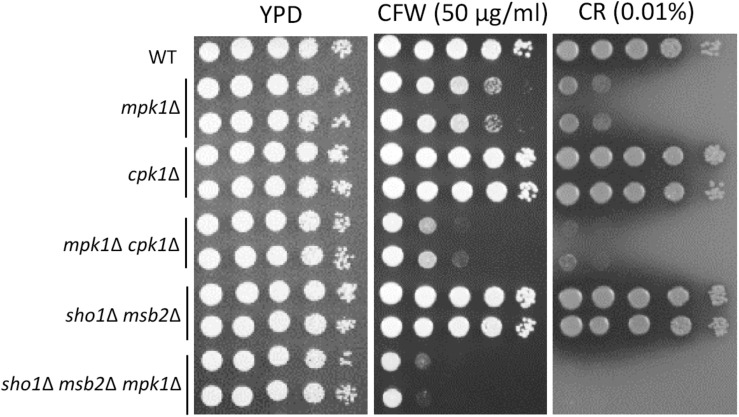
Sho1 and Msb2 have a redundant role in regulating cell wall integrity. Wild-type (WT; H99), *mpk1*Δ (YSB3814 and YSB3816), *cpk1*Δ (YSB127 and YSB128), *mpk1*Δ *cpk1*Δ (YSB6089 and YSB6091), *sho1*Δ *msb2*Δ (YSB3605 and YSB3606), and *sho1*Δ *msb2*Δ *mpk1*Δ (YSB6675 and YSB6676). Each strain was grown overnight at 30°C in YPD medium, 10-fold serially diluted, and spotted onto YPD medium containing the indicated concentrations of Congo red (CR) and calcofluor white (CFW). The plates were further incubated for 2–3 days and photographed. This spot assay was repeated more than three times and one representative image was shown here.

In *Candida albicans*, the Cek1 MAPK, which is orthologous to Cpk1 in *C. neoformans*, is involved in the cell-wall biogenesis (Roman et al., 2009). We assessed whether CPK1 deletion exacerbates the cell-wall integrity defects in cells deleted of Mpk1, which is the cell-wall integrity-regulating MAPK in *C. neoformans*, to prove that Cpk1 is involved in the cell-wall biogenesis. As reported earlier (Kraus et al., 2003), the *mpk1*Δ mutant showed highly increased susceptibility to CFW and CR, whereas the *cpk1*Δ mutant did not (Figure 8). Notably, the *cpk1*Δ *mpk1*Δ mutants showed even more enhanced susceptibility to CFW and CR than the *mpk1*Δ mutants (Figure 8), indicating that Mpk1 and Cpk1 play major and minor roles, respectively, in the cell-wall integrity. To assess the role of Sho1 and Msb2 in the cell-wall integrity, we also constructed the *sho1*Δ *msb2*Δ *mpk1*Δ triple mutants in *C. neoformans*. The *sho1*Δ *msb2*Δ *mpk1*Δ triple mutants were also more susceptible to CFW and CR than the *mpk1*Δ mutants (Figure 8). Collectively, Sho1 and Msb2 contribute to cell wall biogenesis, along with Mpk1 and Cpk1, in *C. neoformans*.

We assessed whether *CPK1* deletion exacerbates the cell-wall integrity defects in cells deleted of Mpk1, which is the cell-wall integrity-regulating MAPK in *C. neoformans* to further prove that Cpk1 is involved in the cell-wall biogenesis. As reported earlier (Kraus et al., 2003), the *mpk1*Δ mutant showed highly increased susceptibility to CFW and CR, whereas the *cpk1*Δ mutant did not (Figure 8B). Notably, the *cpk1*Δ *mpk1*Δ mutants showed even more enhanced susceptibility to CFW and CR than the *mpk1*Δ mutants (Figure 8B). Supporting the redundant role of Sho1 and Msb2 in Cpk1 phosphorylation, the *sho1*Δ *msb2*Δ *mpk1*Δ triple mutants were also more susceptible to CFW and CR than the *mpk1*Δ mutants (Figure 8B). All these results indicated that Mpk1 and Cpk1 play major and minor roles, respectively, in the cell-wall integrity in *C. neoformans*, and Cpk1 is activated by Msb2 and Sho1 for the cell-wall biogenesis.

### Sho1 and Msb2 Play Distinct but Complementary Roles in Pulmonary Virulence of *C. neoformans*

**FIGURE 9:**
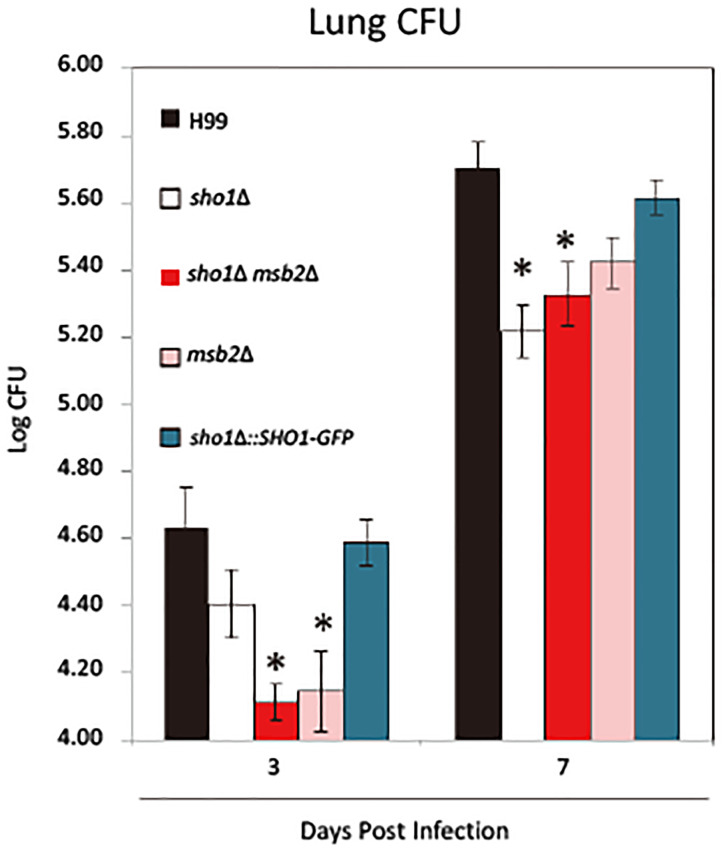
Sho1 and Msb2 contribute to the fungal virulence during *C. neoformans* infection. Mice were infected intratracheally with 10^4^ cells of wild-type (WT; H99), *sho1*Δ (YSB1719), *msb2*Δ (YSB3191), *sho1*Δ *msb2*Δ (YSB3605), and *sho1*Δ::*SHO1-GFP* (YSB2753) strains of *C. neoformans*. Infected lungs were harvested and homogenized for the fungal burden evaluation at 3 and 7 dpi. Significant differences between fungal burdens in the lungs infected with *sho1*Δ, *msb2*Δ, and *sho1*Δ *msb2*Δ versus wild-type strains were found at 3 and (or) 7 dpi. Experiments were repeated independently with similar results (*N* = 6/experimental group or above. ^∗^*P* < 0.05 post-adjustment for multiple comparisons).

Previous studies reported that cryptococcal Sho1 contributes to the pulmonary virulence of *C. neoformans* by its interference with the immune responses (Malachowski et al., 2016). The role of Msb2 and its relationship with Sho1 in virulence during *C. neoformans* infection remains unknown. Using our established model of pulmonary cryptococcosis in mice (Malachowski et al., 2016), we compared the virulence of *sho1*Δ, *msb2*Δ, and *sho1*Δ *msb2*Δ strains *in vivo*. The effects of mutations on the fungal growth in the lungs were analyzed on day 3, illustrating the ability for the fungal adaptation to the host environment and on day 7, which reflects the outcomes of the organism’s interaction with building up pulmonary immune defenses (Hoag et al., 1995). Consistent with our previous study (Malachowski et al., 2016), Sho1 deletion resulted in no marked defect in the pulmonary growth on day 3, relative to H99 and the complement strain. However, we observed a marked growth suppression of *sho1*Δ on day 7 (Figure 9). Conversely, Msb2 deletion resulted in a marked decrease in the fungal burden on day 3, suggesting that Msb2 plays a role in the acute adaptation to the host environment; however, Msb2 seems not to be crucial for the optimal growth of fungus in the lungs thereafter, since the trend for the suppressed fungal burden is no longer significant on day 7. Combined Sho1 and Msb2 deletion (*msb2*Δ *sho1*Δ) did not result in an amplification of a growth defect but showed the effect comparable with the more dominant single-mutant *msb2*Δ on day 3 and *sho1*Δ on day 7, respectively (Figure 9). Overall, the results indicated that although both Msb2 and Sho1 contributed to the pulmonary virulence of *C. neoformans*, Msb2 was particularly important for the early adaptation to the host’s lung environment, whereas Sho1 promoted the growth of fungus during the later time points, characterized by more developed immune defenses (Malachowski et al., 2016). Thus, Sho1 and Msb2 play complementary but distinct roles in the pulmonary virulence of *C. neoformans* during the infection.

## Discussion

This study for the first time proposed the regulatory mechanism of the Sho1-dependent and Msb2-dependent signaling pathways in *C. neoformans*. In addition, this study demonstrated that Sho1 is largely dispensable for the regulation of the HOG pathway for the osmoresistance, thermotolerance, and cryostress resistance. Instead, Sho1 plays Hog1-independent roles in the osmoresistance and thermotolerance. We also found that *C. neoformans* contains Msb2, which is the mucin-like TM Msb2 protein ortholog, known to interact with Sho1 in *S. cerevisiae* (Tatebayashi et al., 2007). However, while *C. neoformans* Msb2 and Sho1 appear to be colocalized in similar subcellular compartments, there is no evidence of their direct interactions. Supporting this, Sho1 and Msb2 play complementary, but distinct roles in biological responses of *C. neoformans*. Like Sho1, Msb2 contributes to the osmotolerance, cell membrane integrity, and cryostress resistance, but frequently not to the same extent, and is not markedly involved in regulation of Hog1 phosphorylation. Sho1 and Msb2 play also overlapping roles in the late stage of sexual differentiation, filamentous growth, in the Cpk1-independent manner. Furthermore, Cpk1, Sho1 and Msb2 contribute to cell wall biogenesis, along with Mpk1. However, during pulmonary infection in the mammalian host cryptococcal Msb2 and Sho1 roles are distinct. Msb2 promotes the acute adaptation to the host environment and seems to be dispensable thereafter. By contrast, Sho1 does not play a substantial role during the acute adaptation but it is required for the optimal fungal growth of fungus in the lungs during the later time points (Figure 9; Malachowski et al., 2016) where, as we demonstrated, it interferes with the development of the immune defenses.

In the model budding yeast *S. cerevisiae*, Sho1 serves as osmosensing adaptor proteins, working with two osmosensor proteins Msb2 and Hkr1, and constitutes one of two signaling branches, along with the Sln1/Ypd1/Ssk1 two-component system, for activation of the Hog1 MAPK stress response pathway (Van Wuytswinkel et al., 2000). However, this study presented several lines of evidence demonstrating that it is not the case in *C. neoformans*. First, the *SHO1* deletion did not affect the phosphorylation status of Hog1 under all tested environmental stresses. Second, the stress-related phenotypic traits observed in the *sho1*Δ, *msb2*Δ, and *sho1*Δ *msb2*Δ mutants were entirely different from those of the HOG pathway mutants. On the basis of our findings demonstrating that Hog1 could be still phosphorylated by the osmotic shock in the *sho1*Δ *ssk1*Δ and *msb2*Δ *ssk1*Δ mutants, a previously uncharacterized signaling branch, other than the two-component phosphorelay system, should exist upstream of the Hog1 MAPK pathway in *C. neoformans*; this upstream pathway warrants further investigation in the future. However, the fact that the osmotic shock-induced Hog1 phosphorylation level was weakly reduced in the *sho1*Δ *ssk1*Δ and *msb2*Δ *ssk1*Δ mutants compared with the *ssk1*Δ mutant indicates that Sho1 and Msb2 may play minor roles in Hog1 phosphorylation in the absence of Ssk1 in *C. neoformans*.

The role of Sho1 in stress sensing and regulation of the Hog1 MAPK pathway seems to be divergent among fungi. In the ascomycete fungal pathogen *C. albicans*, for example, Sho1 plays a minor role in the osmosensitivity in a Hog1-independent manner (Roman et al., 2005). *SHO1* or *SSK1* deletion (or deletion of both) does not affect the osmotic shock-dependent Hog1 phosphorylation (Roman et al., 2005), which is equivalent to the case in *C. neoformans*. Despite the similar minor roles of CaSho1 and CnSho1 in osmosensing, CnSho1 seemingly works differently from CaSho1 in many ways. First, CaSho1 promotes the cellular resistance against oxidative stresses, such as H_2_O_2_ and menadione, mainly in a Hog1-independent manner (Roman et al., 2005). In *Candida lusitaniae* and *Aspergillus fumigatus*, Sho1 orthologs are involved in the oxidative stress response (against H_2_O_2_ and menadione) (Boisnard et al., 2008; Ma et al., 2008). Conversely, CnSho1 is dispensable for resistance to these oxidative damaging agents (Supplementary Figure S3A). Second, CaSho1 is required for the cell-wall biogenesis, as the *Casho1*Δ mutant is highly susceptible to CR and CFW and shows aggregated phenotypes (Roman et al., 2005). By contrast, such phenotypes were not observed in *C. neoformans sho1*Δ mutants (Supplementary Figure S3B). Finally, the role of Sho1 as a temperature sensor appears to be conserved; however, its regulatory mechanism is different among fungi. This study demonstrates that the temperature-upshift stress is sensed by both Ssk1 and Sho1 branches and the temperature downshift primarily sensed by the Ssk1 branch and, in part, by the Msb2 branch in *C. neoformans*. Besides Ssk1 and Sho1, the Hog1 MAPK seemingly uses an additional upstream regulator(s) upon the temperature upshift. In *S. cerevisiae*, however, heat stress and cold stress responses are separately regulated by the Sho1 and Sln1 branches, respectively, upstream of the Hog1 MAPK (Winkler et al., 2002; Hayashi and Maeda, 2006). Overall, the function and regulatory mechanism of Sho1 appear to be evolutionarily divergent among fungi.

The divergent function of Msb2 among fungi was also evident in adaptation to the temperature shift. This study demonstrated that Msb2 was dispensable for thermotolerance but was required for the cryostress response and adaptation in *C. neoformans*; this is in stark contrast to the finding that Msb2 plays a critical role in thermotolerance by regulating the Cek1 MAPK in *C. albicans*. The *C. albicans* *msb2*Δ mutant shows much more severe defects in growth at 37°C–42°C than at 30°C (Saraswat et al., 2016). Although it remains unclear why Msb2 has different functions among fungi, it could be attributed to highly divergent protein sequence among fungal Msb2 orthologs. In *C. albicans*, the extracellular domain of Msb2 is responsible for its function in thermotolerance by regulating the protein kinase C (PKC) pathway (Saraswat et al., 2016). However, a significant sequence homology does not exist between extracellular domains of *C. albicans* and *C. neoformans* Msb2 orthologs. The presence of divergent extracellular domains of Msb2 implies that its function and regulatory mechanism could be divergent among fungi.

Despite the divergent function of Sho1 and Msb2 among fungi, their role in the filamentous growth and morphological differentiation seems evolutionarily conserved, although their regulatory mechanisms are rather different. This study suggests that Sho1 and Msb2 play a redundant role in promoting the filamentous growth of *C. neoformans* but does not regulate pheromone production during mating, which is well-known to be regulated by the Cpk1 MAPK pathway (Kss1 in *S. cerevisiae* and Cek1 in *C. albicans*). In *C. albicans*, however, Sho1 and Msb2 promote the filamentous growth and invasive growth by activating and phosphorylating the Cek1 MAPK (Roman et al., 2005). Likewise, Sho1 ortholog in *C. lusitaniae* is also known to be involved in the pseudohyphal development (Boisnard et al., 2008). In *S. cerevisiae*, Sho1 serves as a receptor for the pseudohyphal growth pathway (O’Rourke and Herskowitz, 1998). In *A. fumigatus*, Sho1 also controls the hyphal development (Ma et al., 2008). In another basidiomycetous fungus, *Ustilago maydis*, Sho1 (UmSho1) also regulates the Cpk1-like MAPKs, Kpp2 and Kpp6, both of which are required for the appressorium development and its function, although UmSho1 is not involved in mating and stress responses, implicating that UmSho1 is uncoupled to the HOG pathway (Lanver et al., 2010).

Although Sho1 and Msb2 do not regulate Cpk1-mediated pheromone production during mating, we found that the two proteins have a redundant role, along with Cpk1 and Mpk1, in regulating the cell-wall integrity in *C. neoformans*. The *cpk1*Δ mutant does not show any increased susceptibility to cell-wall destabilizers, CFW and CR, and an ER stress agent TM (Lee et al., 2016), which is in stark contrast to the *C. albicans cek1*Δ mutant displaying the increased sensitivity to cell-wall and ER stress agents (Roman et al., 2009). This study, however, reported that Cpk1, indeed, plays a minor role in the cell-wall biogenesis of *C. neoformans*, as the *cpk1*Δ *mpk1*Δ and *sho1*Δ *msb2*Δ *mpk1*Δ mutants show a higher cell-wall integrity defect than the *mpk1*Δ mutant; this finding indicates that Cpk1 and Mpk1 play redundant roles in the cell-wall biogenesis in *C. neoformans*, although the latter plays more dominant roles. Thus, this study is the first to report that Cpk1 is involved in the cell-wall biogenesis during the vegetative growth of *C. neoformans*, besides its known role in sexual differentiation.

Regarding the roles of Sho1 and Msb2 in the fungal virulence, prior research has revealed that Sho1 plays a role in promoting immunomodulatory effects of *C. neoformans* and contributes to the fungal growth during pulmonary infection rather than increasing fungal fitness within the host promoting its adaptation to the new environment (Malachowski et al., 2016). This study further supports this conclusion by revealing no marked difference between the WT strain and the *sho1*Δ on day 3, but lower fungal burdens in the *sho1*Δ-infected lungs on day 7 compared with the WT strain. By contrast, *msb2*Δ, as well as *sho1*Δ *msb2*Δ, strains show both early growth defects (day 3), suggesting that Msb2 is critical for the rapid adaptation of *C. neoformans* to the host environment. The outcomes of *sho1*Δ *msb2*Δ double-mutant infections displaying the same level of growth suppression as *msb2Δ* and *sho1*Δ on days 3 and 7, respectively, provide the additional evidence that these two factors affect the fungal virulence independently at each of the selected time points. The differences between the effects of *SHO1* and *MSB2* deletion on the fungal growth in the infected lungs were quite unexpected. While these findings provide an initial evidence of differential role of these genes in the early interactions between the microbe and the mammalian host, the exact mechanisms how they interact with and modify the host defenses remains to be determined in future. Remarkably, in *C. albicans*, besides the sensor function, the extracellular Msb2 domain protected fungal cells effectively from antimicrobial peptides (Szafranski-Schneider et al., 2012). Perhaps, in the early phase of cryptococcal infection (especially before the ingestion by macrophages), the microbe is more susceptible to the action of antimicrobial peptides; however, the molecular mechanisms by which Msb2 contributed to this early adaptation of *C. neoformans* warrants further investigation. In conclusion, this study strongly suggests that Msb2 and Sho1 play distinct roles in the fungal virulence corresponding to the *in vitro* data displaying that Sho1 and Msb2 play distinct roles in stress response/cell-wall integrity and regulating the expression of virulence factor such as capsule in *C. neoformans*.

## Author Contributions

Y-SS, JJ, GP, MO, and YS-B wrote the manuscript. Y-SS, JJ, GP, and JX performed the experiments. Y-SS, JJ, and GP performed the images processing.

## Conflict of Interest Statement

The authors declare that the research was conducted in the absence of any commercial or financial relationships that could be construed as a potential conflict of interest.
