# Corrigendum: Sho1 and Msb2 Play Complementary but Distinct Roles in Stress Responses, Sexual Differentiation, and Pathogenicity of *Cryptococcus neoformans*

**DOI:** 10.3389/fmicb.2020.01956

**Published:** 2020-09-24

**Authors:** Yee-Seul So, Juyeong Jang, Goun Park, Jintao Xu, Michal A. Olszewski, Yong-Sun Bahn

**Affiliations:** ^1^Department of Biotechnology, College of Life Science and Biotechnology, Yonsei University, Seoul, South Korea; ^2^Division of Pulmonary and Critical Care Medicine, Department of Internal Medicine, University of Michigan Medical School, Ann Arbor, MI, United States; ^3^VA Medical Center Ann Arbor Research Service, Ann Arbor, MI, United States

**Keywords:** HOG, mucin, *C. neoformans*, mating, osmotic stress

We recently found that the phospho-p44/42 antibody (Cell Signaling Technology) used in the original article is not specific to the phosphorylated form of Cpk1 in *Cryptococcus neoformans*. Therefore, we removed Cpk1 phosphorylation-related content and Figures (Figures 7D, 8A) and corrected the original article, as shown below.

**Figure 7:**
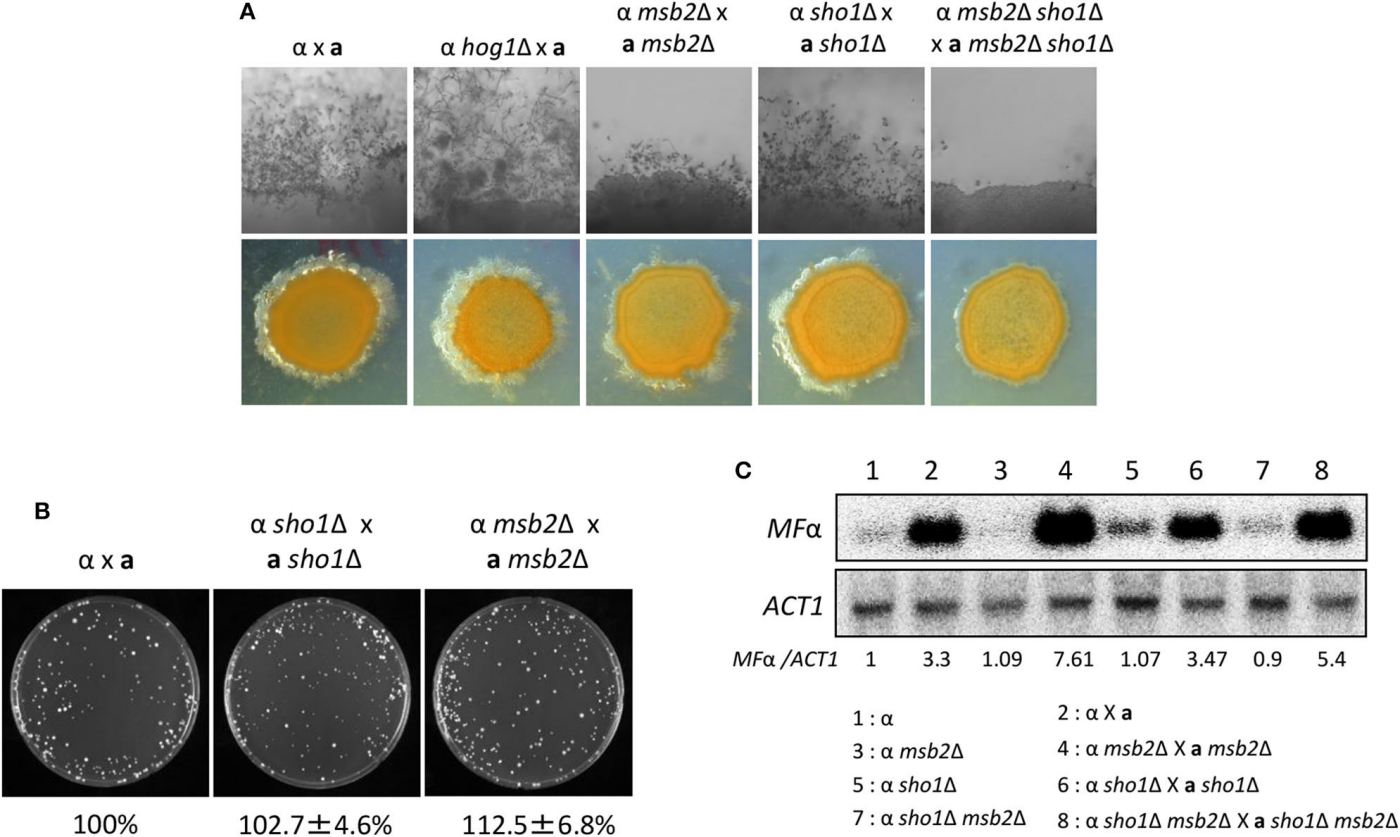
Sho1 and Msb2 play redundant positive roles in the filamentous growth of *C. neoformans*. **(A)** Opposite mating type (*MAT*α and *MAT***a**) cells were incubated for 16 h in YPD liquid medium at 30°C. Opposite mating type cells were mixed at equal concentration (10^7^ cells/mL), spotted (5 μL) on V8 medium, and further incubated in the dark at room temperature for 2 weeks. This mating experiment was repeated twice and one representative image was shown here. **(B)** Mixed opposite mating type cells were spotted on V8 medium and incubated for 1 day at room temperature in the dark. After cells were grown on V8 medium, the cells were resuspended in 1-mL dH_2_O and diluted to 1/100. Then, 200 μL of the suspension was spread on YPD medium containing nourseothricin and G418. The plates were further incubated at 30°C and colonies were counted. **(C)** The northern blot analysis was performed with total RNAs from strains grown on V8 medium for 18 h. The northern blot membrane was hybridized with the mating pheromone-gene (*MF*α*1*)-specific probe. This northern blot analysis was repeated twice and one representative result was shown here.

**Figure 8:**
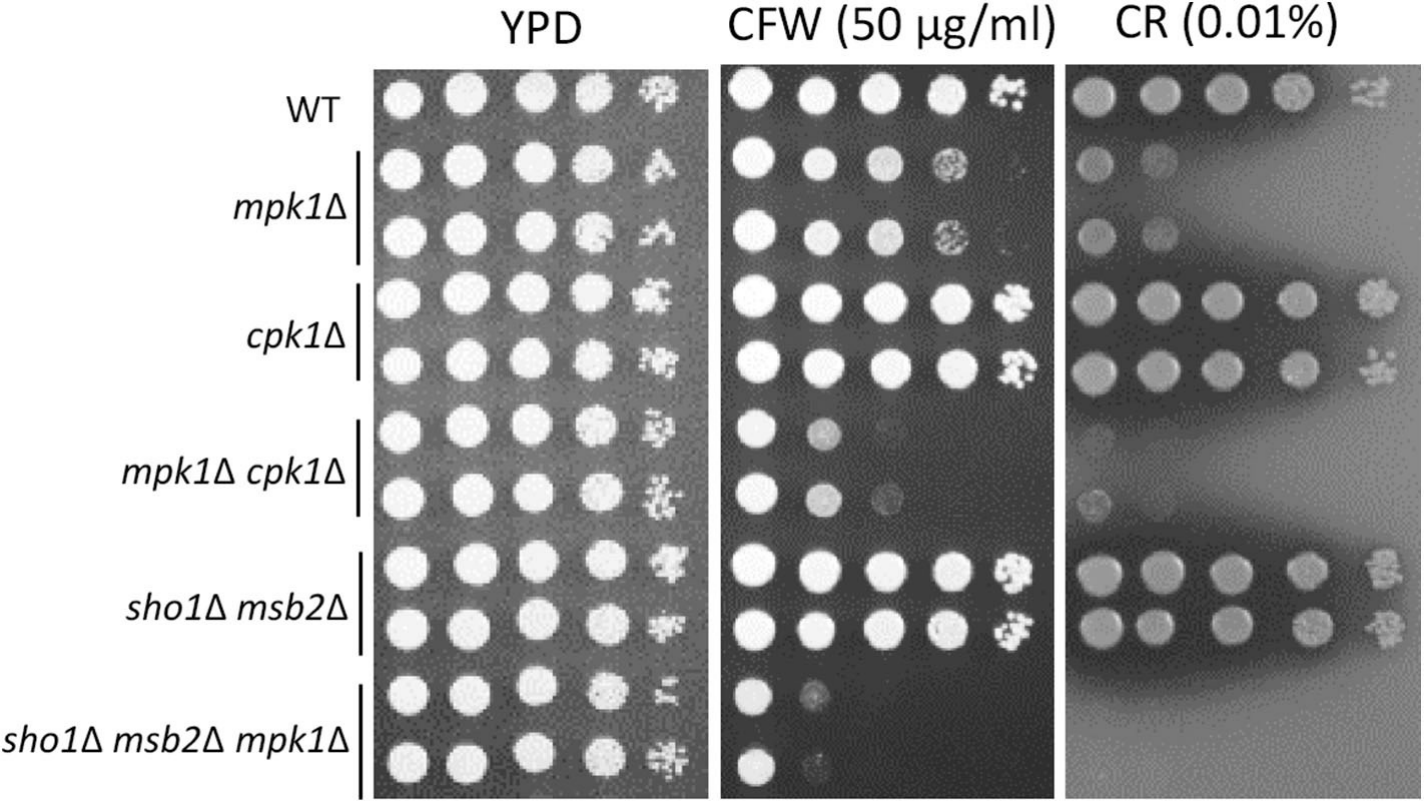
Sho1 and Msb2 have a redundant role in regulating cell wall integrity. Wild-type (WT; H99), *mpk1*Δ (YSB3814 and YSB3816), *cpk1*Δ (YSB127 and YSB128), *mpk1*Δ *cpk1*Δ (YSB6089 and YSB6091), *sho1*Δ *msb2*Δ (YSB3605 and YSB3606), and *sho1*Δ *msb2*Δ *mpk1*Δ (YSB6675 and YSB6676). Each strain was grown overnight at 30°C in YPD medium, 10-fold serially diluted, and spotted onto YPD medium containing the indicated concentrations of Congo red (CR) and calcofluor white (CFW). The plates were further incubated for 2–3 days and photographed. This spot assay was repeated more than three times and one representative image was shown here.

A correction has been made to the **Abstract**.

The high-osmolarity glycerol response (HOG) pathway is pivotal in environmental stress response, differentiation, and virulence of *Cryptococcus neoformans*, which causes fatal meningoencephalitis. A putative membrane sensor protein, Sho1, has been postulated to regulate HOG pathway, but its regulatory mechanism remains elusive. In this study, we characterized the function of Sho1 with relation to the HOG pathway in *C. neoformans*. Sho1 played minor roles in osmoresistance, thermotolerance, and maintenance of membrane integrity mainly in a HOG-independent manner. However, it was dispensable for cryostress resistance, primarily mediated through the HOG pathway. A mucinlike transmembrane (TM) protein, Msb2, which interacts with Sho1 in *Saccharomyces cerevisiae*, was identified in *C. neoformans*, but found not to interact with Sho1. *MSB2* codeletion with *SHO1* further decreased osmoresistance and membrane integrity, but not thermotolerance, of *sho1*Δ mutant, indicating that both factors play to some level redundant but also discrete roles in *C. neoformans*. Sho1 and Msb2 played redundant roles in promoting the filamentous growth in sexual differentiation in a Cpk1-independent manner, in contrast to the inhibitory effect of the HOG pathway in the process. Both factors also played redundant roles in maintaining cell wall integrity in the absence of Mpk1. Finally, Sho1 and Msb2 play distinct but complementary roles in the pulmonary virulence of *C. neoformans*. Overall, Sho1 and Msb2 play complementary but distinct roles in stress response, differentiation, and pathogenicity of *C. neoformans*.

Corrections have been made to the **Materials and Methods** section, sub-section **Western Blot Analysis for the Hog1 Phosphorylation**.

**“Western Blot Analysis for the Hog1 Phosphorylation**.

Each strain was grown in 50-mL YPD medium at 30°C for 16 h. Then, the overnight culture was inoculated into fresh YPD medium and, then, incubated for about 4 h at 30°C to the OD600 of 0.6. A 50 mL of the liquid culture was used at each stress time point. At various time points after the stress, 50 mL of cell suspension was mixed with equal volume of ice-cold stop solution (0.9% NaCl, 1 mM NaN3, 10 mM EDTA, and 50 mM NaF). The cells were harvested at 3000 rpm at 4°C for 5 min and, then, washed once in ice-cold stop solution. The cell pellet was resuspended in the lysis buffer (50-mM Tris–HCl pH 7.5, 1% sodium deoxycholate, 5-mM sodium pyrophosphate, 10-nM sodium orthovanadate, 50-mM NaF, 0.1% SDS, and 1% Triton X-100) containing protease inhibitor cocktail (Calbiochem) and disrupted with 0.5-mm zirconia/silica beads (BioSpec Products, Inc.). After collecting the cell lysates, protein concentrations were determined using the Pierce BCA Protein Assay Kit (Thermo Scientific), and an equal amount of protein was loaded into a 10% SDS-PAGE gel and transferred to Immunoblot PVDF membrane (Bio-Rad). For detecting the phosphorylated forms of Hog1, we used phospho-p38 MAPK antibody (Cell Signaling Technology). In addition, anti-Hog1 antibody (Santa Cruz Biotechnology, SC-2004) was used as a loading control. Secondary antibody used was goat anti-rabbit immunoglobulin G peroxidase-conjugated (Santa Cruz Biotechnology, SC-2004) and the blot was developed using the ECL solution.”

A correction has been made to the **Materials and Methods** section, sub-section **Mating, Cell Fusion, and Pheromone Gene Expression Assay**.

“For analyzing mating phenotypes opposite mating type (*MAT*α and *MAT***a**) cells were cultured in YPD medium at 30°C for 16 h and equal concentration of cells (10^7^ cells/mL) were mixed, spotted onto V8 mating media (pH 5), and incubated in the dark at room temperature for 1–2 weeks. The filamentous growth was monitored and photographed using an Olympus BX51 microscope equipped with a SPOT Insight digital camera. For the cell fusion assay, the concentration of cells was adjusted to 10^7^ cells/mL with phosphate-buffered saline. Each *MAT*α and *MAT***a** strain was mixed in an equal volume, spotted onto a V8 medium, and incubated in the dark at room temperature for 24 h. Then, the cells were scraped, resuspended in 1- mL distilled water, and spread onto YPD medium containing both nourseothricin (100 μg/mL) and G418 (50 μg/mL). The plates were further incubated at 30°C, and the number of colonies was counted. For monitoring the pheromone gene expression, the *MAT*α and KN99**a** strains were mixed with an equal concentration of cells (10^8^ cells/mL), spread onto the V8 medium, and incubated in the dark at room temperature for 18 or 24 h. Then, cells were scraped, pelleted, frozen in liquid nitrogen, and lyophilized overnight for the total RNA isolation, followed by the northern blot analysis with the specific mating pheromone gene (*MF*α*1*)-specific probe.”

A correction has been made to the **Results** section, sub-section **Sho1 and Msb2 Play Redundant Roles in the Filamentation Process of** ***C. neoformans***, Paragraph 2.

We monitored pheromone expression levels under the unilateral and bilateral mating setup among *sho1*Δ, *msb2*Δ, and *sho1*Δ, *msb2*Δ mutants compared with the WT strain to determine which stage of mating is regulated by Sho1 and Msb2. We observed that the pheromone-gene expression was as markedly induced in the *sho1*Δ, *msb2*Δ, and *sho1*Δ *msb2*Δ mutants as WT when α cells were cocultured with **a** cells (Figure 7C). These findings suggested that Sho1 and Msb2 play complementary positive roles in the late stage (filamentation), but not the early stage (pheromone expression and cell fusion), of mating in *C. neoformans*.

The corrected Figure 7 appears below.

Corrections have been made to the **Results** section, sub-section **The Role of Cpk1, Msb2 and Sho1 in the Cell-Wall Integrity of** ***C. neoformans***, Paragraph 1.

**The Role of Cpk1, Msb2 and Sho1 in the Cell-Wall Integrity of** ***C. neoformans***

In *Candida albicans*, the Cek1 MAPK, which is orthologous to Cpk1 in *C. neoformans*, is involved in the cell-wall biogenesis (Roman et al., 2009). We assessed whether *CPK1* deletion exacerbates the cell-wall integrity defects in cells deleted of Mpk1, which is the cell-wall integrity-regulating MAPK in *C. neoformans*, to prove that Cpk1 is involved in the cell-wall biogenesis. As reported earlier (Kraus et al., 2003), the *mpk1*Δ mutant showed highly increased susceptibility to CFW and CR, whereas the *cpk1*Δ mutant did not (Figure 8). Notably, the *cpk1*Δ *mpk1*Δ mutants showed even more enhanced susceptibility to CFW and CR than the *mpk1*Δ mutants (Figure 8), indicating that Mpk1 and Cpk1 play major and minor roles, respectively, in the cell-wall integrity. To assess the role of Sho1 and Msb2 in the cell-wall integrity, we also constructed the *sho1*Δ *msb2*Δ *mpk1*Δ triple mutants in *C. neoformans*. The *sho1*Δ *msb2*Δ *mpk1*Δ triple mutants were also more susceptible to CFW and CR than the *mpk1*Δ mutants (Figure 8). Collectively, Sho1 and Msb2 contribute to cell wall biogenesis, along with Mpk1 and Cpk1, in *C. neoformans*.

The corrected Figure 8 appears below.

A correction has been made to the **Discussion** section, paragraph 1.

“This study for the first time proposed the regulatory mechanism of the Sho1-dependent and Msb2-dependent signaling pathways in *C. neoformans*. In addition, this study demonstrated that Sho1 is largely dispensable for the regulation of the HOG pathway for the osmoresistance, thermotolerance, and cryostress resistance. Instead, Sho1 plays Hog1-independent roles in the osmoresistance and thermotolerance. We also found that *C. neoformans* contains Msb2, which is the mucin-like TM Msb2 protein ortholog, known to interact with Sho1 in *S. cerevisiae* (Tatebayashi et al., 2007). However, while *C. neoformans* Msb2 and Sho1 appear to be colocalized in similar subcellular compartments, there is no evidence of their direct interactions. Supporting this, Sho1 and Msb2 play complementary, but distinct roles in biological responses of *C. neoformans*. Like Sho1, Msb2 contributes to the osmotolerance, cell membrane integrity, and cryostress resistance, but frequently not to the same extent, and is not markedly involved in regulation of Hog1 phosphorylation. Sho1 and Msb2 play also overlapping roles in the late stage of sexual differentiation, filamentous growth, in the Cpk1-independent manner. Furthermore, Cpk1, Sho1 and Msb2 contribute to cell wall biogenesis, along with Mpk1. However, during pulmonary infection in the mammalian host cryptococcal Msb2 and Sho1 roles are distinct. Msb2 promotes the acute adaptation to the host environment and seems to be dispensable thereafter. By contrast, Sho1 does not play a substantial role during the acute adaptation but it is required for the optimal fungal growth of fungus in the lungs during the later time points (Figure 9; Malachowski et al., 2016) where, as we demonstrated, it interferes with the development of the immune defenses.”

A correction has been made to the **Discussion** section, paragraph 5.

“Despite the divergent function of Sho1 and Msb2 among fungi, their role in the filamentous growth and morphological differentiation seems evolutionarily conserved, although their regulatory mechanisms are rather different. This study suggests that Sho1 and Msb2 play a redundant role in promoting the filamentous growth of *C. neoformans* but does not regulate pheromone production during mating, which is well-known to be regulated by the Cpk1 MAPK pathway (Kss1 in *S. cerevisiae* and Cek1 in *C. albicans*). In *C. albicans*, however, Sho1 and Msb2 promote the filamentous growth and invasive growth by activating and phosphorylating the Cek1 MAPK (Roman et al., 2005). Likewise, Sho1 ortholog in *C. lusitaniae* is also known to be involved in the pseudohyphal development (Boisnard et al., 2008). In *S. cerevisiae*, Sho1 serves as a receptor for the pseudohyphal growth pathway (O'Rourke and Herskowitz, 1998). In *A. fumigatus*, Sho1 also controls the hyphal development (Ma et al., 2008). In another basidiomycetous fungus, *Ustilago maydis*, Sho1 (UmSho1) also regulates the Cpk1-like MAPKs, Kpp2 and Kpp6, both of which are required for the appressorium development and its function, although UmSho1 is not involved in mating and stress responses, implicating that UmSho1 is uncoupled to the HOG pathway (Lanver et al., 2010).”

A correction has been made to the **Discussion** section, paragraph 6.

“Although Sho1 and Msb2 do not regulate Cpk1-mediated pheromone production during mating, we found that the two proteins have a redundant role, along with Cpk1 and Mpk1, in regulating the cell-wall integrity in *C. neoformans*. The *cpk1*Δ mutant does not show any increased susceptibility to cell-wall destabilizers, CFW and CR, and an ER stress agent TM (Lee et al., 2016), which is in stark contrast to the *C. albicans cek1*Δ mutant displaying the increased sensitivity to cell-wall and ER stress agents (Roman et al., 2009). This study, however, reported that Cpk1, indeed, plays a minor role in the cell-wall biogenesis of *C. neoformans*, as the *cpk1*Δ *mpk1*Δ and *sho1*Δ *msb2*Δ *mpk1*Δ mutants show a higher cell-wall integrity defect than the *mpk1*Δ mutant; this finding indicates that Cpk1 and Mpk1 play redundant roles in the cell-wall biogenesis in *C. neoformans*, although the latter plays more dominant roles. Thus, this study is the first to report that Cpk1 is involved in the cell-wall biogenesis during the vegetative growth of *C. neoformans*, besides its known role in sexual differentiation.

The authors apologize for these errors and state that this does not change the main scientific conclusions of the article. The original article has been updated.
