# Supplementary material for: Sho1 and Msb2 Play Complementary but Distinct Roles in Stress Responses, Sexual Differentiation, and Pathogenicity of Cryptococcus neoformans
Source: Front Microbiol. 2018 Dec 4;9:2958. doi: 10.3389/fmicb.2018.02958 (PMC6288190; doi:10.3389/fmicb.2018.02958)
Supplement: Supplementary file 1 [file Data_Sheet_1.pdf]

Supplementary Table S1. *C. neoformans* strains used in this study

Strain	Genotype	Parent	Reference
H99	*MAT*α		(Perfect *et al.*, 1993)
KN99	*MAT***a**		(Nielsen *et al.*, 2003)
YSB64	*MAT*α *hog1*Δ*::NAT-STM#177*	H99	(Bahn *et al.*, 2005)
YSB123	*MAT*α *pbs2*Δ*::NAT-STM#213*	H99	(Bahn *et al.*, 2005)
YSB261	*MAT*α *ssk1*Δ*::NAT-STM#205*	H99	(Bahn *et al.*, 2006)
YSB264	*MAT*α *ssk2*Δ*::NAT-STM#210*	H99	(Bahn *et al.*, 2007)
YSB349	*MAT*α *skn7*Δ*::NAT-STM#201*	H99	(Bahn *et al.*, 2006)
YSB278	*MAT*α *tco1*Δ*::NAT-STM#102*	H99	(Bahn *et al.*, 2006)
YSB281	*MAT*α *tco2*Δ*::NAT-STM#116*	H99	(Bahn *et al.*, 2006)
YSB324	*MAT*α *tco1*Δ*::NAT-STM#102 tco2*Δ*::NEO*	YSB278	(Bahn *et al.*, 2006)
YSB284	*MAT*α *tco3*Δ*::NAT-STM#119*	H99	(Bahn *et al.*, 2006)
YSB417	*MAT*α *tco4*Δ*::NAT-STM#123*	H99	(Bahn *et al.*, 2006)
YSB286	*MAT*α *tco5*Δ*::NAT-STM#125*	H99	(Bahn *et al.*, 2006)
YSB2469	*MAT*α *tco6*Δ*::NAT-STM#58*	H99	(Lee *et al.*, 2016)
YSB348	*MAT*α *tco7*Δ*::NAT-STM#209*	H99	(Bahn *et al.*, 2006)
YSB1719	*MAT*α *sho1*Δ*::NAT-STM#58*	H99	(Kim *et al.*, 2015)
YSB1720	*MAT*α *sho1*Δ*::NAT-STM#58*	H99	(Kim *et al.*, 2015)
YSB1927	*MAT***a** *sho1*Δ*::NEO*	KN99	This study
YSB1928	*MAT***a** *sho1*Δ*::NEO*	KN99	This study
YSB3191	*MAT*α *msb2*Δ*::NAT-STM#150*	H99	This study
YSB3916	*MAT***a** *msb2*Δ*::NEO*	KN99	This study
YSB3605	*MAT*α *msb2*Δ*::NAT-STM#150 sho1*Δ*::NEO*	YSB3191	This study
YSB3606	*MAT*α *msb2*Δ*::NAT-STM#150 sho1*Δ*::NEO*	YSB3191	This study
YSB3945	*MAT***a** *msb2*Δ*::NEO sho1*Δ*::NAT-STM#58*	YSB3916	This study
YSB3592	*MAT*α *SHO1-6×HA*	H99	This study
YSB3593	*MAT*α *SHO1-6×HA*	H99	This study
YSB3631	*MAT*α *MSB2-4×FLAG*	H99	This study
YSB3698	*MAT*α *SHO1-6×HA MSB2-4×FLAG*	YSB3593	This study
YSB2253	*MAT*α *ssk1*Δ*::NAT-STM#205 sho1*Δ*::NEO*	YSB261	This study
YSB2268	*MAT*α *hog1*Δ*::NAT-STM#177 sho1*Δ*::NEO*	YSB64	This study
YSB2753	*MAT*α *sho1*Δ*::SHO1-GFP*	YSB1719	(Malachowski *et al.*, 2016)
YSB3455	*MAT*α *MSB2-mCherry*	H99	This study
YSB4132	*MAT*α *sho1*Δ*::SHO1-GFP MSB2-mCherry*	YSB2753	This study
YSB3814	*MAT*α *mpk1*Δ*::NAT*	H99	(Lee *et al.*, 2016)
YSB3816	*MAT*α *mpk1*Δ*::NAT*	H99	(Lee *et al.*, 2016)
YSB127	*MAT*α *cpk1*Δ*::NAT*	H99	(Lee *et al.*, 2016)
YSB128	*MAT*α *cpk1*Δ*::NAT*	H99	(Lee *et al.*, 2016)
YSB6089	*MAT*α *mpk1*Δ*::NAT cpk1*Δ*::NEO*	YSB3814	This study
YSB6091	*MAT*α *mpk1*Δ*::NAT cpk1*Δ*::NEO*	YSB3814	This study
YSB6674	*MAT*α *msb2*Δ*::NAT-STM#150 ssk1*Δ*::HYG*	YSB3191	This study
YSB6675	*MAT*α *msb2*Δ*::NAT-STM#150 sho1*Δ*::NEO mpk1*Δ*::HYG*	YSB3606	This study
YSB6676	*MAT*α *msb2*Δ*::NAT-STM#150 sho1*Δ*::NEO mpk1*Δ*::HYG*	YSB3606	This study

Each *NAT-STM#* indicates the Nat^r marker with a unique signature tag.

Bahn, Y. S., S. Geunes-Boyer and J. Heitman (2007) Ssk2 mitogen-activated protein kinase kinase kinase governs divergent patterns of the stress-activated Hog1 signaling pathway in *Cryptococcus neoformans*. Eukaryot. Cell **6**: 2278-2289.
Bahn, Y. S., K. Kojima, G. M. Cox and J. Heitman (2005) Specialization of the HOG pathway and its impact on differentiation and virulence of *Cryptococcus neoformans*. Mol. Biol. Cell **16**: 2285-2300.
Bahn, Y. S., K. Kojima, G. M. Cox and J. Heitman (2006) A unique fungal two-component system regulates stress responses, drug sensitivity, sexual development, and virulence of *Cryptococcus neoformans*. Mol. Biol. Cell. **17**: 3122-3135.
Kim, H., K. W. Jung, S. Maeng, Y. L. Chen, J. Shin, J. E. Shim*, et al.* (2015) Network-assisted genetic dissection of pathogenicity and drug resistance in the opportunistic human pathogenic fungus Cryptococcus neoformans. Sci Rep **5**: 8767.
Lee, K. T., Y. S. So, D. H. Yang, K. W. Jung, J. Choi, D. G. Lee*, et al.* (2016) Systematic functional analysis of kinases in the fungal pathogen Cryptococcus neoformans. Nat Commun **7**: 12766.
Malachowski, A. N., M. Yosri, G. Park, Y. S. Bahn, Y. He and M. A. Olszewski (2016) Systemic Approach to Virulence Gene Network Analysis for Gaining New Insight into Cryptococcal Virulence. Front Microbiol **7**: 1652.
Nielsen, K., G. M. Cox, P. Wang, D. L. Toffaletti, J. R. Perfect and J. Heitman (2003) Sexual cycle of Cryptococcus neoformans var. grubii and virulence of congenic a and alpha isolates. Infection and immunity **71**: 4831-4841.
Perfect, J. R., N. Ketabchi, G. M. Cox, C. W. Ingram and C. L. Beiser (1993) Karyotyping of Cryptococcus neoformans as an epidemiological tool. J Clin Microbiol **31**: 3305-3309.

Supplementary Table S2. Primers used in this study

Primer Name	Sequence (5’ to 3’)	comments
B79	TGTGGATGCTGGCGGAGGATA	Screening primer
B1026	GTAAAACGACGGCCAGTGAGC	M13Forward primer
B1027	CAGGAAACAGCTATGACCATG	M13Reverse primer
B1454	AAGGTGTTCCCCGACGACGAATCG	*NAT* PCR primer SM1
B1455	AACTCCGTCGCGAGCCCCATCAAC	*NAT* PCR primer SM2
B1886	TGGAAGAGATGGATGTGC	*NEO* Split GSR
B1887	ATTGTCTGTTGTGCCCAG	*NEO* Split GSL
B5751	CGAAGAATCTCGTGCTTTC	HSM (Hygromycin Split Marker) 1
B5752	ATTGACCGATTCCTTGCG	HSM2
B4204	ATCTCCCAATCTCCCGAAG	*SHO1* PCR primer L1
B4205	TCACTGGCCGTCGTTTTACAAGAAAGACTGGGTGTCGC	*SHO1* PCR primer L2
B4206	CATGGTCATAGCTGTTTCCTGACACCCGCTGGTATTACAG	*SHO1* PCR primer R1
B4207	AAGTTTTCCTCCACTCGCC	*SHO1* PCR primer R2
B4203	AACGAACGGAAGATTGGC	*SHO1* PCR primer SO
B4208	GCTGCTTACTACATCTGGACG	*SHO1* PCR primer PO
B4504	CGACACCCAGTCTTTCTTG	*SHO1* northern blot probe primer 1
B4505	GTAATACCAGCGGGTGTTC	*SHO1* northern blot probe primer 2
B4794	GTCGACATCTCCCAATCTCCCGAAG	*sho1*Δ::*SHO1* primer L1
B4795	GCGGCCGCTCACTTATCGTCGTCATCCTTGTAATCAAGCAAGACAAGGTAATTGG	*sho1*Δ::*SHO1* primer R1
B4796	GCGGCCGCGACTAAAATGATGACTTGAAC	*sho1*Δ::*SHO1* primer L2
B4797	CCGCGGAAGTTTTCCTCCACTCGCC	*sho1*Δ::*SHO1* primer R2
B4998	GCTGCTTACTACATCTGGACG	Sho1::GFP primer seq1
B5614	GCGGCCGCATCTCCCAATCTCCCGAAG	Sho1::GFP primer L1
B5610	GCGGCCGCAAGCAAGACAAGGTAATTGGAG	Sho1::GFP primer L2
B6144	TCACCTACACAGCAGACCAC	*MSB2* PCR primer L1
B6145	CTGGCCGTCGTTTTACATGAAGGCGGTGTGTATCG	*MSB2* PCR primer L2
B6146	GTCATAGCTGTTTCCTGGCGTTGAGTAAGCCTAAGC	*MSB2* PCR primer R1
B6147	GAAGAGATGACGGGAGTTATG	*MSB2* PCR primer R2
B6148	AATCTTCGTCCAGTTGTCAC	*MSB2* PCR primer SO
B6395	AGAACTGTTTGAGAAGGCG	*MSB2* PCR primer PO2
B6724	CGCAACATCATAATCGGTG	*MSB2* FLAG PCR primer L1
B6729	GGCTGCTTTTGACAATGG	*SHO1* HA PCR primer L1
B6732	TAATCGCTGAGCCGTTTG	*SHO1* HA PCR primer SO
B6900	CATTCAAACCGCCTCTTC	*MSB2* mCherry PCR primer L1
B6901	CTCCTCGCCCTTGCTCACAGAGCCACCGCCACCTGCGTG	*MSB2* mCherry PCR primer L2
B6902	TGCCACTCGAATCCTGCATGCGACGTGAGACTCGACGG	*MSB2* mCherry PCR primer R1
B6991	CCGTTCAGAATCATACAACC	*MSB2* FLAG PCR primer PO
JOHE12039	CTGTAGAAGATGTGAGTTTGGG	*CPK1* PCR primer L1
JOHE12040	CTGGCCGTCGTTTTACTGATTGATGAGAGATACGGG	*CPK1* PCR primer L2
JOHE12041	GTCATAGCTGTTTCCTGGGCGGAGAAATAGAGGTTG	*CPK1* PCR primer R1
JOHE12042	CGCACAAGAAGTAAGAGGTG	*CPK1* PCR primer R2
JOHE12043	GGCTATGGACCGTATTCAC	*CPK1* PCR primer SO
JOHE12046	ATGCTGCTCACCGTTAGTC	*CPK1* PCR primer PO
B5944	CCGATTTGGAAGTCGTTG	*SSK1* PCR primer L1
B5945	CTGGCCGTCGTTTTACAATCAAAGAGAGGCTGGG	*SSK1* PCR primer L2
B5946	GTCATAGCTGTTTCCTGTCTCCATTTCGCTCGTCAG	*SSK1* PCR primer R1
B5947	GCAAACTCCAAAAACGCTC	*SSK1* PCR primer R2
B5948	GAAACCTTGTCGCTGAGAC	*SSK1* PCR primer SO
B3668	GCAGAGGTTAGAGGAAGTC	*SSK1* PCR primer PO
B5930	TTTGCTTGCTCCTCTTCTC	*MPK1* PCR primer L1
B5931	TCACTGGCCGTCGTTTTACGAGAAGTAGAGGCAGTGACG	*MPK1* PCR primer L2
B5932	CATGGTCATAGCTGTTTCCTGTTGGAGAAACAGTTGGAGAG	*MPK1* PCR primer R1
B5933	TTCAGCAGGTCAATCAGG	*MPK1* PCR primer R2
B5934	CGACTCACGATGTAACTTCC	*MPK1* PCR primer SO
B5935	ACCTCAACTCTCTCAGACACC	*MPK1* PCR primer PO

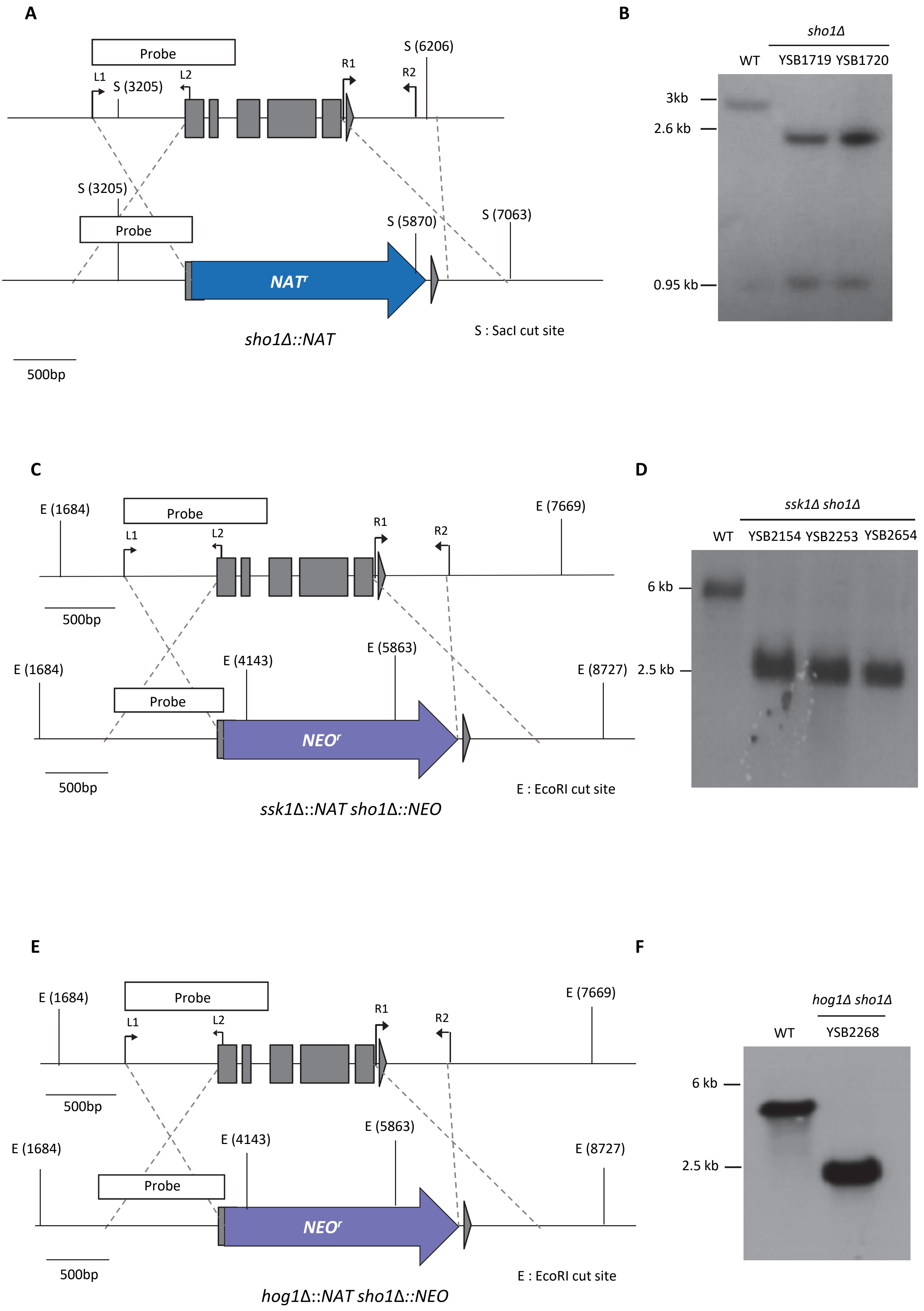
A
Probe
L1
S (3205)
L2
R1
S (6206)
R2
S (3205)
Probe
S (5870)
S (7063)
NAT^r
sho1Δ::NAT
S : SacI cut site
500bp
B
sho1Δ
WT
YSB1719 YSB1720
3kb
2.6 kb
0.95 kb
C
E (1684)
Probe
E (7669)
L1
L2
R1
R2
500bp
E (1684)
E (4143)
E (5863)
E (8727)
Probe
NEO^r
500bp
E : EcoRI cut site
ssk1Δ::NAT sho1Δ::NEO
D
ssk1Δ sho1Δ
WT
YSB2154 YSB2253 YSB2654
6 kb
2.5 kb
E
E (1684)
Probe
E (7669)
L1
L2
R1
R2
500bp
E (1684)
E (4143)
E (5863)
E (8727)
Probe
NEO^r
500bp
E : EcoRI cut site
hog1Δ::NAT sho1Δ::NEO
F
hog1Δ sho1Δ
WT
YSB2268
6 kb
2.5 kb

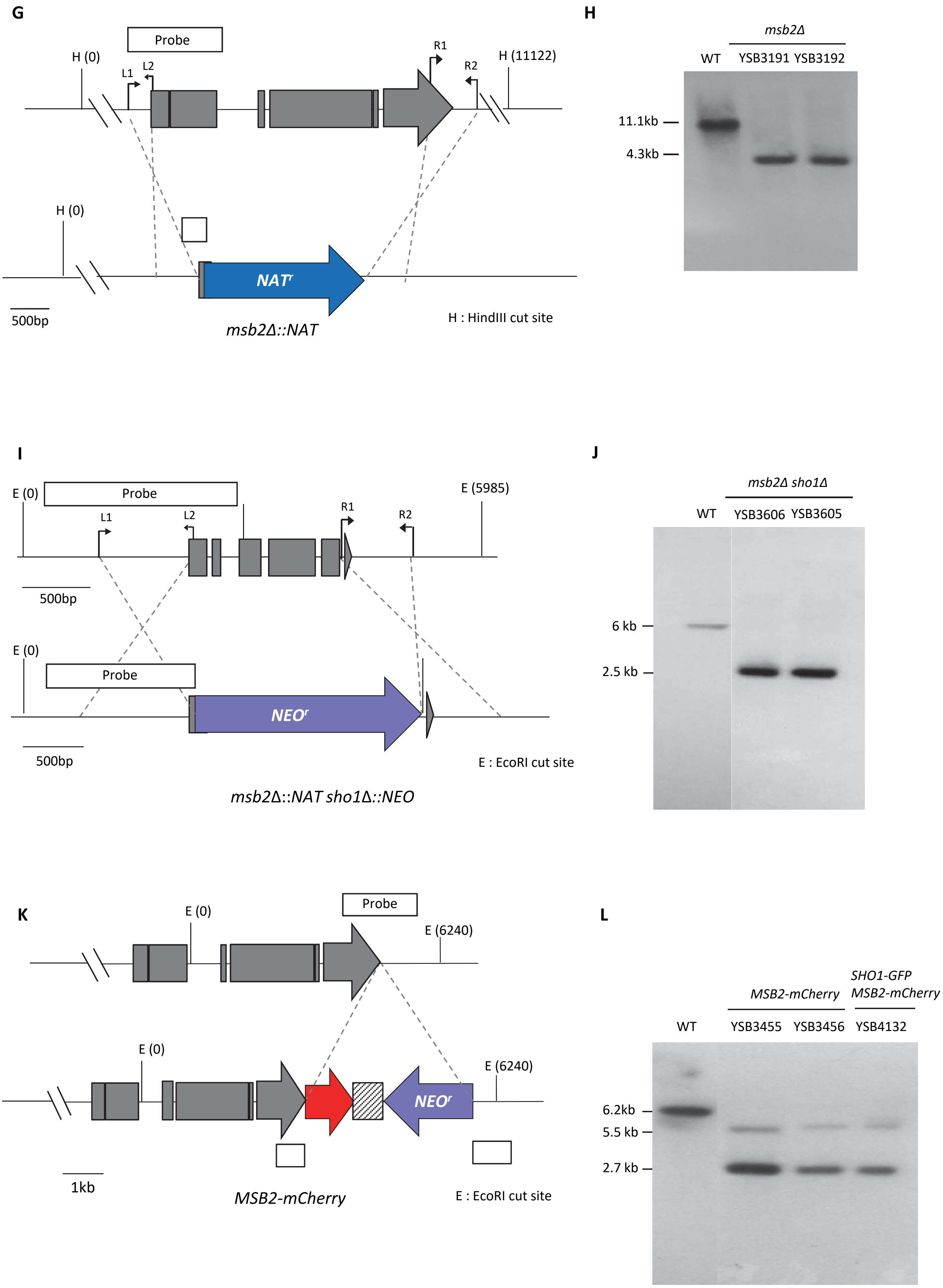
G
Probe
H (0)
L1
L2
R1
R2
H (11122)
H (0)
NAT^r
500bp
msb2Δ::NAT
H : HindIII cut site
H
msb2Δ
WT
YSB3191
YSB3192
11.1kb
4.3kb
I
E (0)
Probe
L1
L2
R1
R2
E (5985)
500bp
E (0)
Probe
NEO^r
500bp
E : EcoRI cut site
msb2Δ::NAT sho1Δ::NEO
J
msb2Δ sho1Δ
WT
YSB3606
YSB3605
6 kb
2.5 kb
K
E (0)
Probe
E (6240)
E (0)
E (6240)
NEO^r
1kb
MSB2-mCherry
E : EcoRI cut site
L
SHO1-GFP
MSB2-mCherry
MSB2-mCherry
WT
YSB3455
YSB3456
YSB4132
6.2kb
5.5 kb
2.7 kb

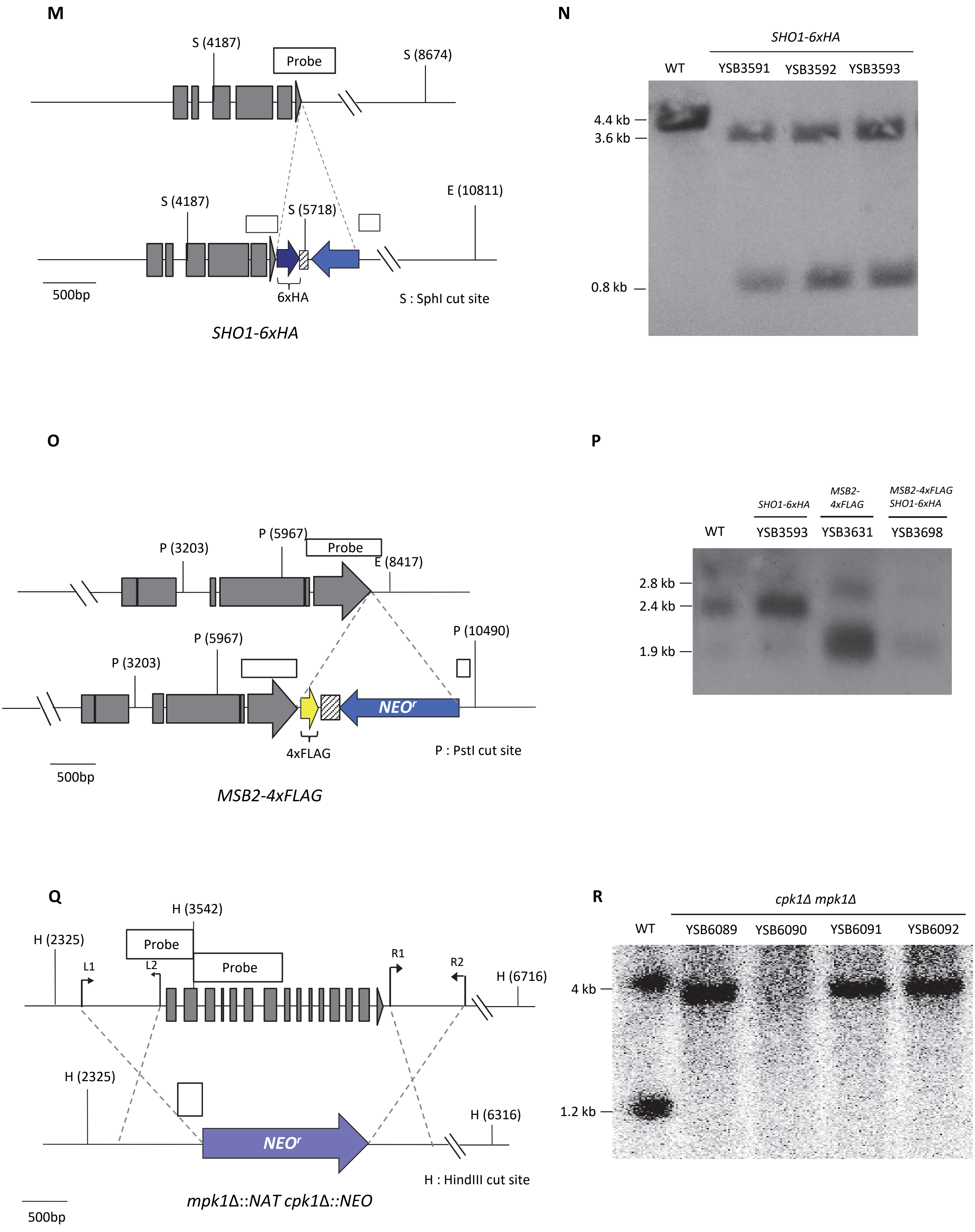

M
S (4187)
Probe
S (8674)
S (4187)
S (5718)
E (10811)
500bp
6xHA
S : SphI cut site
SHO1-6xHA
N
SHO1-6xHA
WT
YSB3591
YSB3592
YSB3593
4.4 kb
3.6 kb
0.8 kb
O
P (3203)
P (5967)
Probe
E (8417)
P (5967)
P (10490)
P (3203)
NEOr
4xFLAG
P : PstI cut site
500bp
MSB2-4xFLAG
P
SHO1-6xHA
MSB2-4xFLAG
MSB2-4xFLAG SHO1-6xHA
WT
YSB3593
YSB3631
YSB3698
2.8 kb
2.4 kb
1.9 kb
Q
H (3542)
H (2325)
Probe
Probe
L1
L2
R1
R2
H (6716)
H (2325)
H (6316)
NEOr
H : HindIII cut site
mpk1Δ::NAT cpk1Δ::NEO
500bp
R
cpk1Δ mpk1Δ
WT
YSB6089
YSB6090
YSB6091
YSB6092
4 kb
1.2 kb

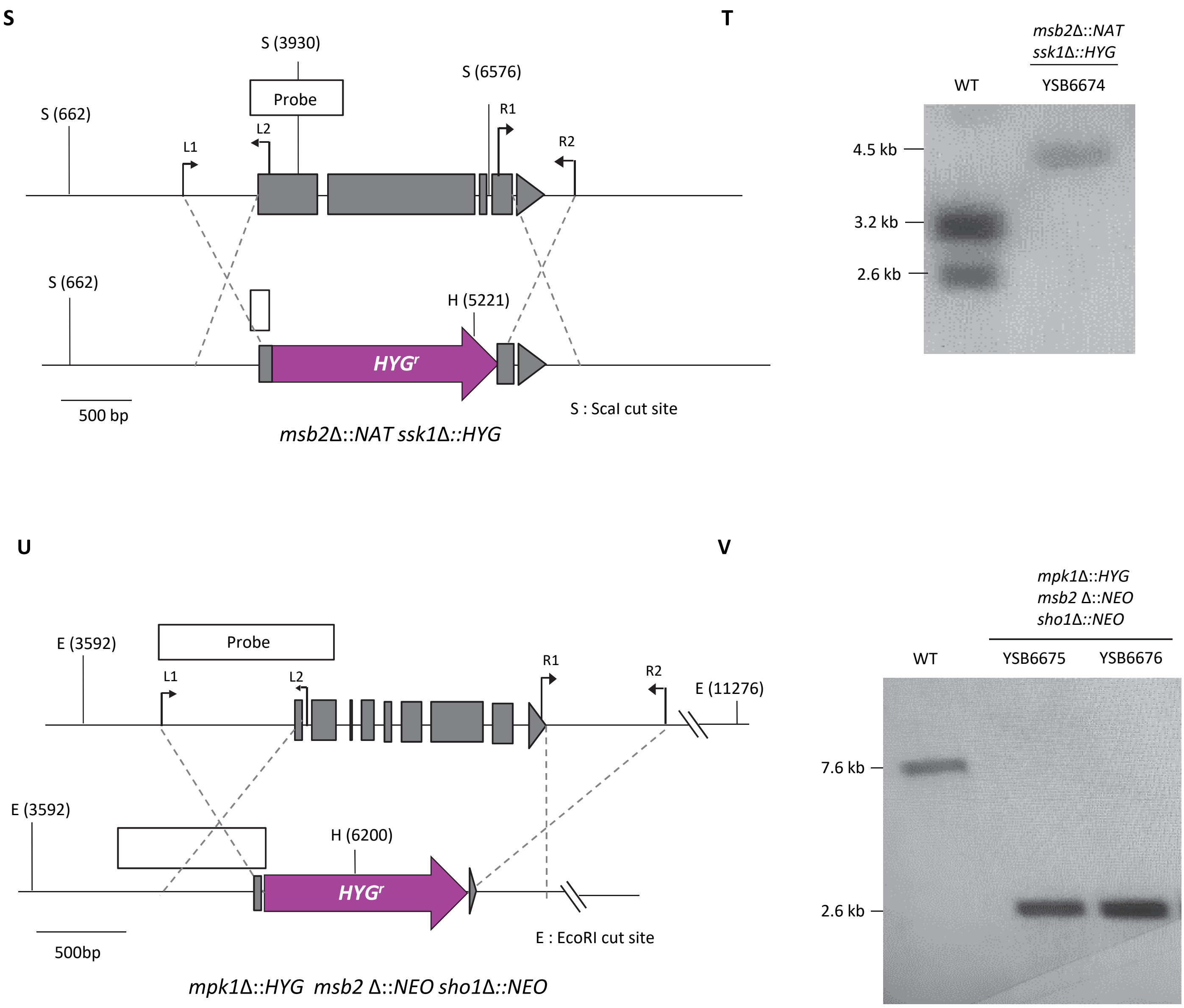

Supplementary Fig. S1. Construction of tagged or deletion strains. (A, C, E, G, I, K, M, O, Q, S and U) Schematic strategy for the construction of tagged or deletion strains. (B, D, F, H, J, L, N, P, R, T and V) The correct genotypes of the strains were confirmed by Southern blot analysis.

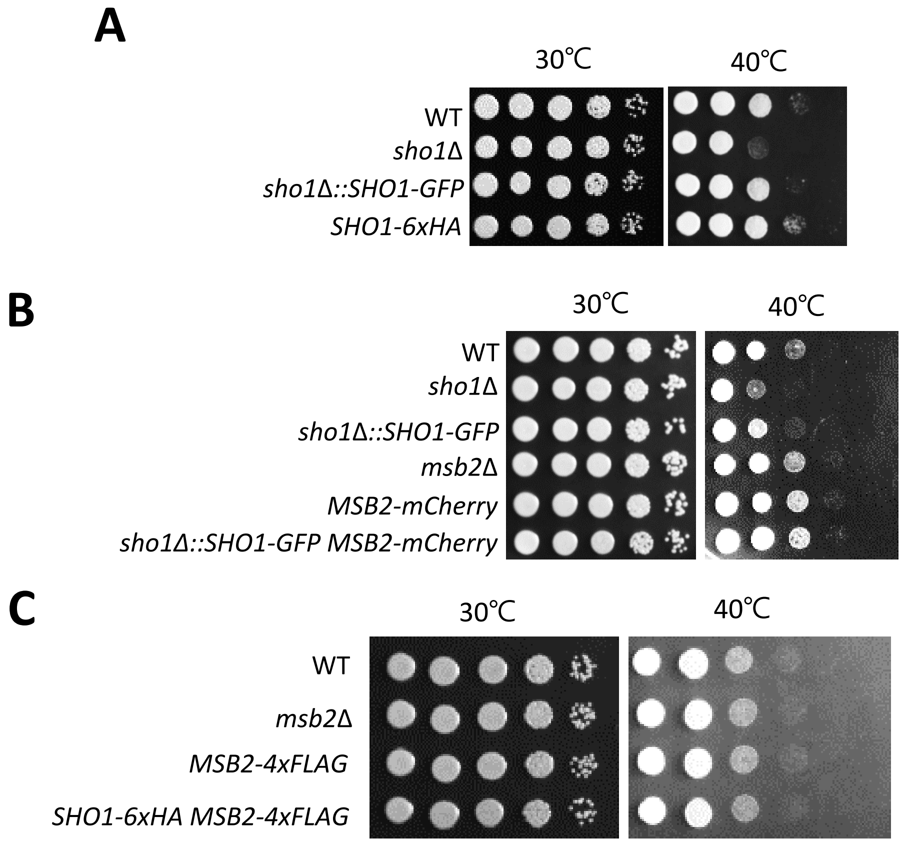

Supplementary Figure S2. Phenotypic analyses of tagged strains. *C. neoformans* strains were cultured overnight in liquid YPD medium at 30°C, and then the cultures were spotted on YPD medium. The spotted cells were incubated at 30°C or 40°C. [Strains: WT (H99), *sho1*Δ (YSB1719), *sho1Δ::SHO1-GFP* (YSB2753), *SHO1-6xHA* (YSB3593), *msb2*Δ (YSB3191),*MSB2-mCherry* (YSB4128), *sho1Δ::SHO1-GFP MSB2-mCherry* (YSB4132), *MSB2-4xFLAG* (YSB3631), *SHO1-6xHA MSB-4xFLAG* (YSB3698)]

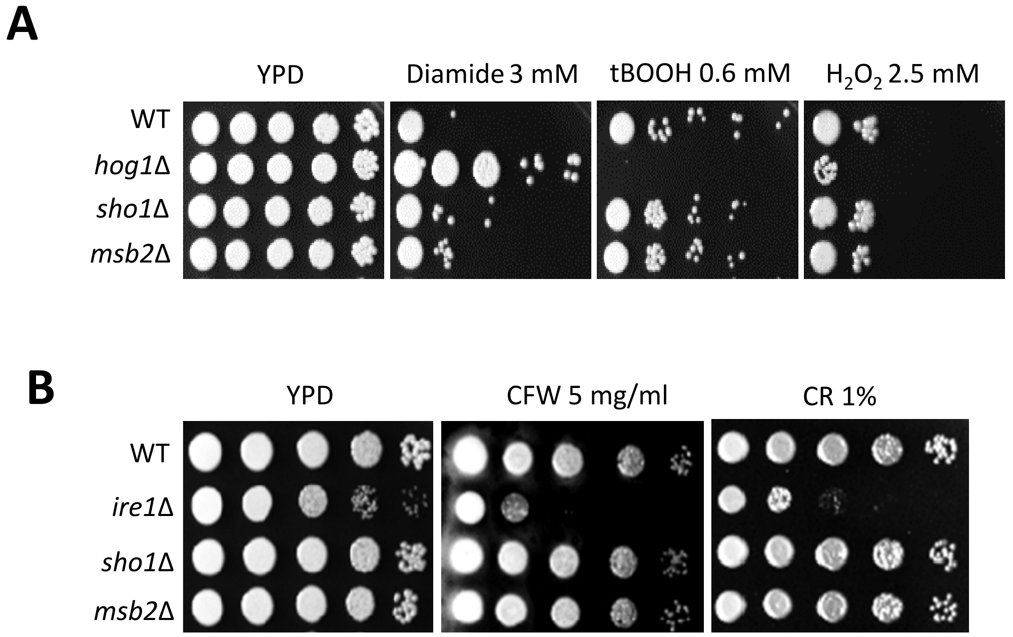

Supplementary Fig. S3. Sho1 and Msb2 are dispensable for resistance to oxidative damaging agents and cell wall destabilizers. Strains were grown overnight at 30℃ in liquid YPD medium. The strains were serially diluted and spotted 3μl on YPD medium containing the Diamide, tBOOH, H_2O_2, CFW (calcofluor white)or CR (conco red). The plates were incubated at 30℃ for 2 days and photographed (A-B).
